# A Crown Ether-Ammonium Supramolecular Platform for Luminescent [Cu_4_I_6_]^2–^ Cluster Phosphors

**DOI:** 10.1021/acsphyschemau.6c00098

**Published:** 2026-07-24

**Authors:** Disnel Ferrera-Carracedo, Vojtech Jancik, Estrella Ramos, Diego Solis-Ibarra, Juan de Dios Guzmán-Hernández, J. C. Alonso-Huitron, Ioantani D. Álvarez, Jesús U. Balderas-Aguilar

**Affiliations:** † Instituto de Investigaciones en Materiales, Universidad Nacional Autónoma de México, Ciudad Universitaria, Ciudad de México 04510, México; ‡ Instituto de Química, Universidad Nacional Autónoma de México, Ciudad Universitaria, Ciudad de México, 04510, México; § Centro Conjunto de Investigación en Química Sustentable UAEM-UNAM, CarreteraToluca-Atlacomulco km. 14.5, Toluca, Estado de México 50200, México

**Keywords:** Crown ether-ammonium, supramolecular assembly, phosphor converter, copper(**I**) iodide cluster, blue-light excitation

## Abstract

Control over the supramolecular environment of emissive metal halide clusters offers an attractive route to tune the photophysical behavior of hybrid phosphors. Among these systems, ionic [Cu_4_I_6_]^2–^ cluster phosphors are promising blue excitable emitters that exhibit broadband cluster-centered luminescence; however, rational strategies for modulating their local environment remain limited. Here, a supramolecular host–guest approach was employed to stabilize and tune [Cu_4_I_6_]^2–^ clusters using 18-crown-6 (18C6) in combination with three ammonium–derived cations of increasing steric and hydrophobic character (NH_4_^+^, CH_3_NH_3_^+^ (MA), and C_8_H_12_N^+^ (PEA)). This design enabled systematic variation of the organic framework while preserving the same emissive cluster motif. Structural analysis, combined with Hirshfeld surface mapping, revealed that the cluster’s structural distortion is governed by the topology and spatial distribution of intermolecular contacts arising from crystal packing. Dense and anisotropic I···H and Cu···H contacts in NH_4_(18C6) and PEA­(18C6) imposed uneven constraints on the [Cu_4_I_6_]^2–^ units, increasing I–Cu–I bond angle distortion and strengthening exciton–phonon coupling, resulting in broader emission. In contrast, MA­(18C6) exhibited fewer host–cluster contacts but significant I···I intercluster interactions, leading to reduced lattice relaxation and narrower photoluminescence. Density functional theory calculations confirmed strong electronic confinement within the [Cu_4_I_6_]^2–^ clusters and revealed that variations in band dispersion arise from differences in the supramolecular environment. Among the series, PEA­(18C6) combined high PLQY (∼80%), improved environmental stability, and efficient blue–driven white light emission. These results demonstrate that supramolecular host–guest design provides a modular platform for tuning structure property relationships in copper halide cluster phosphors and for developing stable, lead free materials for solid–state lighting.

## Graphical abstract

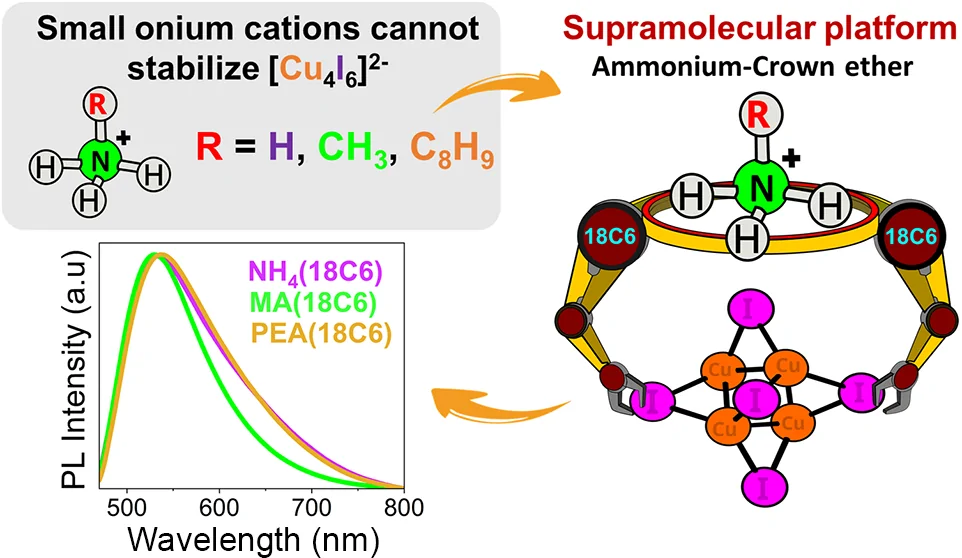

## Introduction

Lead-free hybrid metal halides are a diverse class of compositionally tunable optoelectronic materials that have emerged as environmentally friendly alternatives to hybrid lead halide perovskites.^1,2^ A defining feature of this materials class is its structural and compositional versatility, which enables systematic modification of the inorganic and organic components to tune optoelectronic properties, stability, and processability.^3^ These materials have demonstrated promising performance in applications including down-conversion phosphors for solid-state lighting,^4–7^ scintillators for X-ray imaging,^8–10^ anticounterfeiting technologies,^8,11^ and solar concentrators.^12,13^

Among them, hybrid copper­(I) halides have gained increasing attention owing to their environmental friendliness, low cost, and earth abundance.^14^ The highly electron-rich d^10^ configuration of copper­(I) favors the formation of low-coordinate complexes, typically linear [CuX_2_]^−^, trigonal-planar [CuX_3_]^2–^, or tetrahedral [CuX_4_]^3–^ coordination units (X = Cl, Br, I). Furthermore, these coordination units can connect through face-, edge-, or corner-sharing arrangements to generate larger anionic motifs such as [Cu_2_X_4_]^2–^, [Cu_4_X_6_]^2–^, and [Cu_6_X_9_]^3–^. These motifs generally adopt zero-dimensional configurations, spatially separated by large organic cations,^15^ which promotes strong electronic confinement and efficient luminescence.

The [Cu_4_I_6_]^2–^ cluster, consisting of four trigonal-planar [CuI_3_]^2–^ units connected by corner-sharing, is particularly attractive for phosphor converter materials in solid-state lighting. Compounds featuring these motifs typically exhibit absorption in the violet-blue region and broad emission arising from Cu···Cu metallophilic interactions, giving rise to a cluster-centered (^3^CC) emission.^16^ These clusters self-assemble when the CuI:A^+^I^–^ ratio equals 2:1 (where A is a monovalent cation), and to date, stabilization of these units has primarily relied on bulky organic cations such as alkyltriphenylphosphonium derivatives.^5,17^ When smaller cations such as Cs^+^ or CH_3_NH_3_^+^ are employed, stabilization of the discrete 0D [Cu_4_I_6_]^2–^ cluster becomes unfavorable, and the system instead tends to form extended 1D Cu­(I) iodide frameworks composed of edge-sharing [CuI_4_]^3–^ tetrahedra.^18,19^ Such low-dimensional chain structures exhibit markedly different electronic dimensionality and anisotropic carrier transport characteristics associated with the extended Cu–I connectivity. An alternative strategy to stabilize these [Cu_4_I_6_]^2–^ clusters with smaller cations involves supramolecular encapsulation using crown ethers coordinated to alkali or alkaline-earth cations.^4,6,10^ However, not all crown ether complex cations are suitable for cluster stabilization, and these approaches often provide limited control over the local organic environment surrounding the cluster, sometimes leading to motifs such as linear [CuI_2_]^−^ units or [Cu_2_I_4_]^2–^ dimers. Moreover, systematic strategies capable of independently modulating the supramolecular environment surrounding the [Cu_4_I_6_]^2–^ core, while preserving the cluster topology itself remain scarce.

In this context, supramolecular approaches based on onium-type cations offer a largely unexplored route to modulate the local environment of [Cu_4_I_6_]^2–^ clusters. Onium cations capable of establishing directional noncovalent interactions with halometalate clusters are particularly attractive building blocks for this purpose. These include alkylammonium and aromatic ammonium cations bearing π-conjugated systems, which can promote hydrogen bonding and π···π intermolecular interactions. Collectively, these interactions influence the crystal packing and the local environment surrounding the metal halide clusters, thereby affecting the structural and photophysical properties of the resulting materials. The chemical diversity of ammonium-derived cations, combined with host–guest interactions such as crown ether coordination, provides a modular framework in which the inorganic cluster can be preserved while the surrounding organic environment is systematically varied. Such control provides a rational route to modulate lattice distortion, intermolecular contact topology, exciton–phonon coupling, and ultimately the photophysical response and environmental stability. During the preparation of this work, two independent reports demonstrated chiral amine–crown ether stabilized [Cu_4_I_6_]^2–^ phosphors for circularly polarized luminescence, highlighting the emerging potential of supramolecular design in advanced photonic applications.^20,21^

Herein, the family of ionic [Cu_4_I_6_]^2–^ cluster-based materials is expanded via a supramolecular self-assembly strategy involving 18-crown-6 (18C6) and different achiral onium cations (ammonium, methylammonium, and phenethylammonium), forming the organic framework. This approach yields three new compounds containing the same copper iodide cluster and macrocyclic host, while varying only the size and character of the onium cation to systematically tune organic framework-cluster interactions. Structural and Hirshfeld surface analyses reveal that the topology and spatial distribution of intermolecular contacts govern cluster distortion. More asymmetric I···H and Cu···H contacts in PEA­(18C6) increase I–Cu–I bond angle distortion, leading to stronger exciton–phonon coupling and broader photoluminescence. In contrast, MA­(18C6) exhibits negligible hydrogen contacts but significant I···I intercluster interactions, leading to distinct decay dynamics and reduced PLQY. Electronic structure calculations based on DFT reveal strong confinement of the electronic edge states within the [Cu_4_I_6_]^2–^ clusters, together with increased band dispersion in MA­(18C6), where I···H and Cu···H contacts are negligible, and I···I intercluster interactions dominate. Finally, PEA­(18C6), obtained via a solvent-free mechanochemical route, is demonstrated as a promising phosphor converter for pc-WLEDs. These results establish supramolecular host–guest control as an effective design strategy to tune structure–property relationships in copper halide cluster phosphors.

## Experimental Section

### Materials and Reagents

Copper­(I) iodide (CuI, 98%); 1,4,7,10,13,16-Hexaoxacyclooctadecane (18-crown-6, 99%); ammonium iodide (NH_4_I, 99%); methylammonium iodide (CH_3_NH_3_I, 99%); phenethylammonium iodide (C_8_H_12_NI, 99%); acetone (technical grade) and hypophosphorous acid (H_3_PO_2_, 50 wt % in water) were purchased from Sigma-Aldrich and used without further purification.

### Synthesis of Single Crystals

Ammonium iodide (NH_4_I, 2 mmol), 18-crown-6 (1 mmol), and copper­(I) iodide (CuI, 1 mmol) were dissolved in acetone (6 mL). Subsequently, hypophosphorous acid (H_3_PO_2_, 1 mL) was added to prevent the oxidation of Cu­(I). The resulting solution was stirred until complete dissolution was achieved, followed by filtration using a PTFE filter with 0.44 μm pore size. After approximately 12 h, single crystals suitable for further characterization were obtained by slow evaporation of the solvent at a constant temperature of 30 °C. MA­(18C6) and PEA­(18C6) were synthesized following the same procedure as NH_4_(18C6), using equimolar (1:1:1) amounts of the corresponding precursors.

### Structural Characterization

Single-crystal data were collected using a Bruker Smart Apex Duo diffractometer equipped with an Apex II CCD detector and an Incoatec IμS microfocus source (Mo–Kα radiation, λ = 0.71073 Å) at 100 K. Data acquisition was performed using ω-scan techniques. The collected frames were integrated with SAINT and corrected for absorption effects using SADABS.^22^ The crystal structures were solved by direct methods employing SHELXT^23^ and subsequently refined by full-matrix least-squares on F^2^ using SHELXL-2019/3 ^24^ within the ShelXle graphical interface.^25^ All non-hydrogen atoms were refined anisotropically, while hydrogen atoms were positioned in idealized geometries. Powder X-ray diffraction (PXRD) patterns were recorded on an Ultima IV Rigaku diffractometer using Cu–Kα radiation (λ = 1.5418 Å) over a 2θ range of 5°–50° with a step size of 0.02°.

### Optical and Photophysical Characterization

UV–vis spectroscopy was performed using a Shimadzu UV-2600 spectrophotometer operating in reflectance mode over the 200–800 nm wavelength range. Photoluminescence excitation (PLE) and emission (PL) spectra were recorded on an Edinburgh Instruments FS5 spectrofluorometer equipped with a 150 W ozone-free xenon arc lamp and Czerny-Turner monochromators. Time-resolved photoluminescence (TRPL) and photoluminescence quantum yield (PLQY) measurements were carried out using the same instrument. For TRPL measurements, a picosecond pulsed diode laser (λ = 375 ± 5 nm) was employed as the excitation source, while PLQY measurements were conducted using an integrating sphere. Temperature-dependent measurements were performed on a Fluoromax 3 spectrofluorometer employing a cryostat with helium as a cooling gas.

### Computational Methodology

Electronic structure calculations were performed using density functional theory (DFT) as implemented in the CASTEP code^26^ within the Materials Studio package.^27^ The Perdew–Burke–Ernzerhof (PBE) functional^28^ within the generalized gradient approximation (GGA) was employed, including Tkatchenko–Scheffler dispersion corrections.^29^ A plane-wave cutoff energy of 750 eV and reciprocal-space pseudopotentials were used, including scalar relativistic effects within the Koelling–Harmon approximation.^30^ Calculations were carried out in a spin-polarized scheme with a 2 × 1 × 1 Monkhorst–Pack *k*-point grid.^29^ Geometry optimizations were performed using the BFGS algorithm with convergence criteria of 5 × 10^–5^ eV/atom for total energy, 0.05 eV Å^–1^ for forces, and 0.005 Å for atomic displacements. Electronic self-consistency was achieved with an energy tolerance of 10^–5^ eV using Pulay density mixing and Gaussian smearing of 0.1 eV. Band structure and density of states were obtained from the optimized geometries, and the bandgap was determined from the energy difference between the valence band maximum (VBM) and conduction band minimum (CBM) to support the experimental analysis. Hirshfeld surface analysis and associated fingerprint plots were generated using Crystal Explorer 21.5.^31^ For all structures, disordered components were removed, and only the major occupancy positions were retained. Time-dependent density functional theory (TD-DFT) calculations were performed using Gaussian 16 on the optimized ground-state geometry of the [Cu_4_I_6_]^2–^ cluster coordinated to one ammonium crown ether ligand.^32^ Single point calculations were carried out employing the ωB97X-D exchange-correlation functional and the Def2-TZVP basis set.^33,34^ The lowest excited state (S_1_) was analyzed using Natural Transition Orbitals (NTOs), which provide a compact representation of the electron–hole pair associated with the electronic excitation. The spatial distributions of the hole and electron were further examined using the hole–electron analysis module implemented in Multiwfn,^35^ allowing the visualization of the electron density depletion (hole) and accumulation (electron) associated with the excitation in real space.

### Stability Measurements

Thermogravimetric analysis (TGA) was performed using a Q5000 IR TGA system (TA Instruments, New Castle, DE, USA) under nitrogen flow. Single crystals of the samples were heated from 30 to 600 °C at a rate of 10 °C·min^–1^. Temperature-dependent PXRD measurements were carried out using the same diffractometer employed for room-temperature characterization, equipped with a variable-temperature stage.

### pc-WLED Fabrication and Characterization

For pc-WLED fabrication, PEA­(18C6) powders were first synthesized by mixing equimolar amounts of phenethylammonium iodide, 18-crown-6, and CuI via a mechanochemical route without the use of H_3_PO_2_. The precursors were ground in an agate mortar for approximately 5 min until a homogeneous powder was obtained. Subsequently, the as-prepared powder was dispersed in a polymer matrix and coated onto a 450 nm blue LED chip. For the characterization of the pc-WLED, an LMS-9000C high precision CCD spectroradiometer equipped with an integrating sphere was employed.

## Results and Discussion

### Supramolecular Assembly and Structural Evolution of the [Cu_4_I_6_]^2–^ Cluster Environment

The [Cu_4_I_6_]^2–^ clusters in the three compounds are stabilized through supramolecular self-assembly between 18-crown-6 (18C6) and ammonium-based cations, namely NH_4_^+^, methylammonium (CH_3_NH_3_^+^, MA), or phenethylammonium (C_8_H_12_N^+^, PEA). This strategy provides a modular framework in which the copper halide cluster is preserved while the surrounding supramolecular environment is tuned through the identity of the onium cation. Single crystals were obtained by slow solvent evaporation from acetone solutions containing CuI, 18C6, and the corresponding amine salt. Hypophosphorous acid (H_3_PO_2_) was added to suppress oxidation of copper­(I) during crystallization (Figure 1a). This approach yielded millimeter-sized crystals exhibiting bright photoluminescence under blue light irradiation (450 nm) (Figure S1a–c).

**Figure 1:**
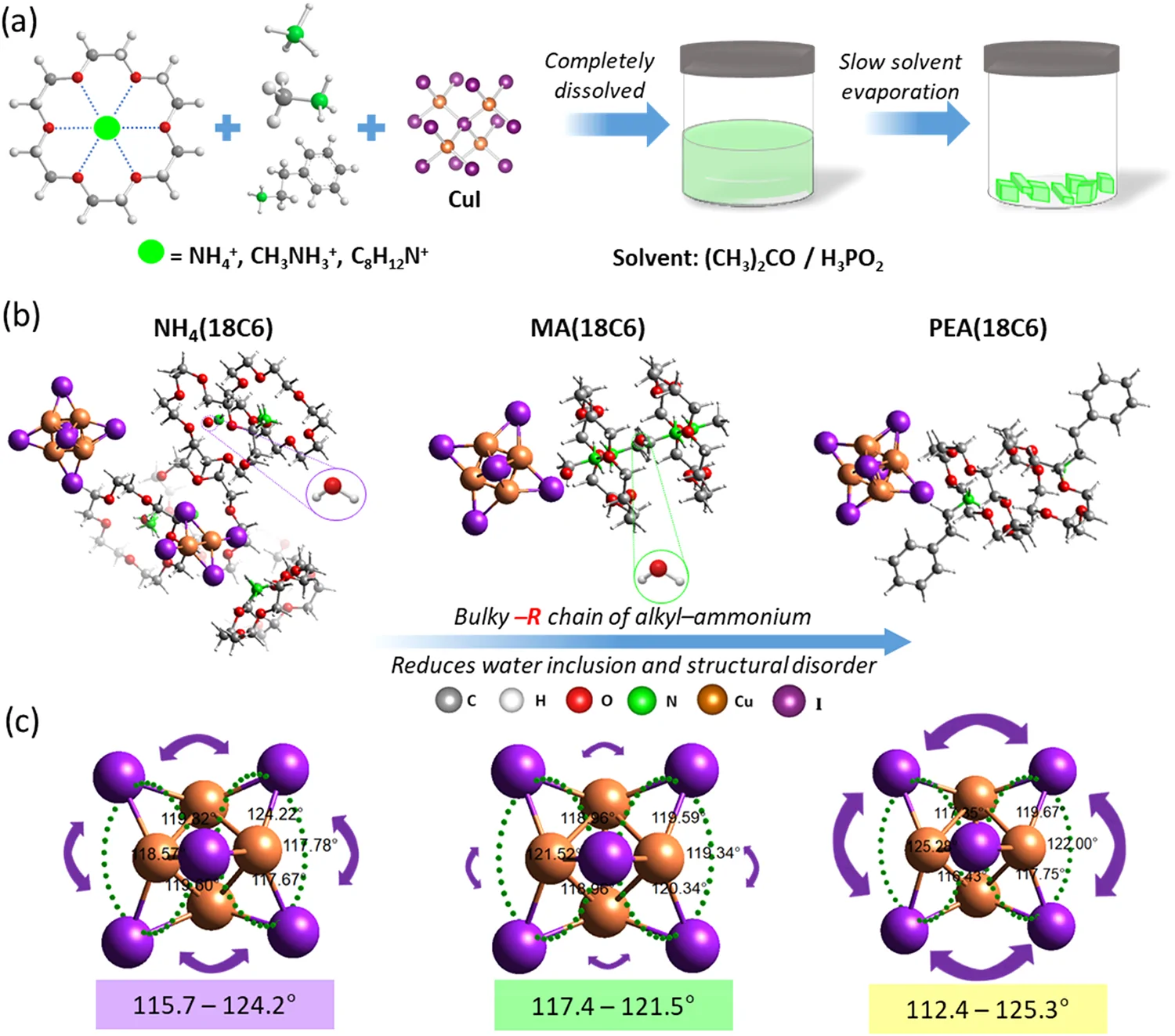
(a) Schematic synthetic procedure for the three obtained compounds. (b) Structural comparison of NH_4_(18C6), MA­(18C6), and PEA­(18C6) showing that increasing alkylammonium steric bulk reduces water inclusion and structural disorder. (c) [Cu_4_I_6_]^2–^ clusters with the range of I–Cu–I bond angle variation for each compound, from left to right NH_4_(18C6), MA­(18C6), and PEA­(18C6).

Detailed structural insights were obtained from the SCXRD analysis. NH_4_(18C6) crystallizes in the monoclinic space group *Pn* as an inversion twin with chemical formula [(NH_4_)_4_(H_2_O)_0.5_(18C6)_6_]­[(Cu_4_I_6_)­(Cu_2_I_4_)]. In addition to the targeted [Cu_4_I_6_]^2–^ cluster, the structure contains a [Cu_2_I_4_]^2–^ dimer (Figure S2a). These two copper­(I) anionic units form a zero-dimensional framework surrounded by highly disordered 18C6 molecules. The crowns interact with disordered NH_4_^+^ cations through N–H···O hydrogen bonding, while water molecules partially occupy competing coordination sites (Figures 1b and S4) with low site occupancies. Although NH_4_(18C6) partially deviates from the targeted single-cluster motif by incorporating a [Cu_2_I_4_]^2–^ dimer, this result is itself informative, as it highlights the limited ability of the smallest and least hydrophobic cation to impose selective supramolecular stabilization of the [Cu_2_I_4_]^2–^ unit when combined with 18C6 crown ether.

To probe how incremental changes in the supramolecular cationic environment modulate cluster organization, hydration, and photophysics, the cation size was increased from NH_4_^+^ to MA and then to PEA. The MA­(18C6) crystallizes in the monoclinic space group *P*2_1_/*c* with formula [(CH_3_NH_3_)_2_(H_2_O)_0.33_(18C6)_2_]­[(Cu_4_I_6_)]. In this case, only the [Cu_4_I_6_]^2–^ motif is present, giving a zero-dimensional structure in which the cluster is isolated by 18C6 molecules hydrogen-bonded to methylammonium (Figure S2b). The 18C6 molecules are fully ordered; however, the CH_3_NH_3_^+^ cations are disordered over two sites (82%:18% occupancy), each located on opposite sides of the crown. A partially occupied water molecule is associated with each MA orientation, approximately coinciding with the methyl group of the alternative configuration (Figures 1b and S5).

Further increasing the steric bulk, the phenethylammonium analogue PEA­(18C6) was synthesized. PEA­(18C6) also crystallizes in the monoclinic space group *P*2_1_/*c* with chemical formula [(C_8_H_12_N)_2_(18C6)_2_]­[Cu_4_I_6_], preserving isolated [Cu_4_I_6_]^2–^ clusters within a zero–dimensional structure (Figure S2c). In contrast with the previous compounds, no structural disorder is observed on the organic complex, and no water molecules are incorporated (Figures 1b and S6). This behavior is attributed to the increased steric volume and hydrophobic character of the phenethyl group. Whereas NH_4_^+^ lacks hydrophobic shielding and readily accommodates water, and MA provides only limited hydrophobic protection through a single −CH_3_ group, PEA effectively excludes water from the supramolecular environment.

The experimental powder X–ray diffraction (PXRD) patterns of NH_4_(18C6), MA­(18C6), and PEA­(18C6) match well with those simulated from SCXRD data, confirming the phase purity of the as-synthesized compounds (Figure S3a–c).

For the three supramolecular compounds, the Cu···Cu separations within the [Cu_4_I_6_]^2–^ clusters are shorter than the sum of the van der Waals radii of Cu­(I) (2.80 Å), indicating significant cuprophilic interactions.^3,36^ The Cu···Cu distances span 2.682–2.768 Å for NH_4_(18C6), 2.717–2.773 Å for MA­(18C6), and 2.721–2.793 Å for PEA­(18C6), with average values of 2.734, 2.739, and 2.753 Å, respectively. Further crystallographic details are provided in the Supporting Information (Tables S1–S3). Another important structural variable across the series is the degree of distortion of the [Cu_4_I_6_]^2–^ cluster. In particular, the I–Cu–I bond angles deviate from the ideal D_3_h symmetry expected for trigonal planar [CuI_3_]^2–^ units. In NH_4_(18C6), these angles range from 115.69° to 124.20°; in MA­(18C6), they fall within a narrower interval (117.36°–121.52°); whereas in PEA­(18C6), they span a broader range, from 112.36° to 125.28° (Figure 1c). These results indicate that the supramolecular cation-crown framework modulates the local geometry of the inorganic cluster and, therefore, provides a structural handle for tuning its photophysical behavior.

### Steady State Photoluminescence as a Function of Supramolecular Environment

Room-temperature steady-state photoluminescence excitation (PLE) and emission (PL) spectra were recorded to demonstrate the intrinsic optical response of the three phosphors (Figure 2a–c). All compounds display broad excitation bands centered at 450 nm and broadband emission spanning the green to red spectral region with extended long wavelength tails, satisfying the requirements for blue light driven phosphor-converter applications.^37,38^

**Figure 2:**
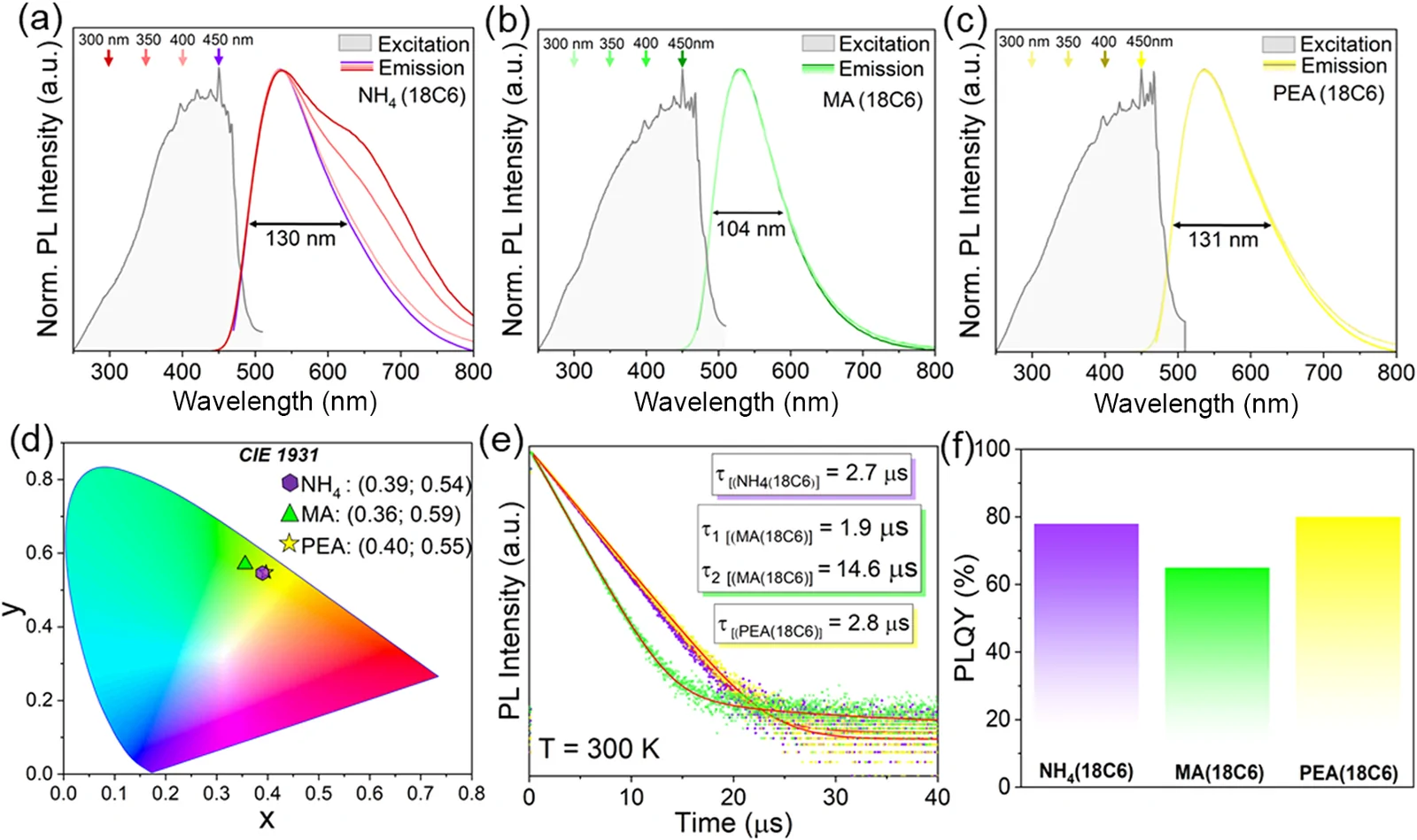
(a–c) Photoluminescence excitation (PLE) and emission (PL) spectra of the three compounds at room temperature. (d) CIE 1931 chromaticity diagram showing the emission color coordinates. (e) Photoluminescence decay curves and corresponding lifetimes; and (f) photoluminescence quantum yields (PLQYs).

NH_4_(18C6) exhibits an emission maximum at 534 nm with a full width at half-maximum (FWHM) of 130 nm under 450 nm excitation. Increasing excitation energy reveals an additional low-energy emission band, consistent with the presence of [Cu_2_I_4_]^2–^ dimers in the structure.^39,40^ Such dimers are known to support broadband visible emission under UV excitation, with absorption features typically centered near 320 nm. The coexistence of [Cu_2_I_4_]^2–^ units and [Cu_4_I_6_]^2–^ clusters therefore introduces multiple emissive centers, leading to excitation-dependent luminescence in NH_4_(18C6) (Figure 2a).

In contrast, MA­(18C6) displays excitation-independent emission, indicative of a single emissive excited state. The emission maximum is slightly blue-shifted to 529 nm, with a narrower FWHM of 104 nm (Figure 2b). PEA­(18C6) likewise exhibits excitation-independent PL behavior, but shows red-shifted emission at 536 nm and a broader FWHM of 131 nm (Figure 2c).

The broader emission observed in NH_4_(18C6) and PEA­(18C6) relative to MA­(18C6) suggests enhanced structural relaxation in the excited state, likely arising from stronger or more asymmetric host-cluster interactions in these two compounds. Similar correlations between structural flexibility and emission bandwidth have been reported in hybrid Mn^2+^ halide-based systems.^41^ The resulting spectral shifts and line width changes translate directly into distinct chromaticity coordinates in the CIE 1931 diagram (Figure 2d), underscoring the sensitivity of cluster-centered excited state to the supramolecular environment.

To elucidate the emissive dynamics and efficiencies of the supramolecular phosphors, room-temperature time-resolved photoluminescence (TRPL) measurements and photoluminescence quantum yield (PLQY) determinations were performed. Decay profiles were collected at the respective emission maxima under 365 nm pulsed excitation (Figure 2e). NH_4_(18C6) and PEA­(18C6) exhibit monoexponential decay with lifetimes of 2.7 and 2.8 μs, respectively, consistent with ^3^CC emission typically observed in copper­(I) iodide [Cu_4_I_6_]^2–^ hybrid compounds.^4,17^

In contrast, MA­(18C6) displays biexponential decay with lifetimes of 1.9 and 14.6 μs, revealing distinct excited-state dynamics. The emergence of a long-lived component suggests an additional relaxation pathway, possibly associated with subtle differences in local symmetry, intermolecular coupling, or intercluster interactions; however, further analysis is required to unambiguously identify its origin.

The PLQYs are 78%, 65%, and 80% for NH_4_(18C6), MA­(18C6), and PEA­(18C6), respectively (Figure 2f). The reduced quantum yield of MA­(18C6) correlates with its complex decay behavior, indicating that the long-lived state likely introduces additional nonradiative losses.

To investigate the excited states and further elucidate the emission mechanism of these compounds, Time Dependent Density Functional Theory (TD-DFT) calculations were performed for MA­(18C6) and PEA­(18C6). Charge Density Difference (CDD) maps were used to analyze the electronic redistribution associated with the first singlet excited state (S_1_), taking into account all electronic excitation contributions (Figure S7a,b). These maps reveal that, for both compounds, the electron density redistribution remains localized within the [Cu_4_I_6_]^2–^ cluster, with no significant charge transfer to the surrounding organic environment. These results indicate that the excited state is predominantly localized on the inorganic cluster, supporting a cluster-centered emission mechanism.

### Intermolecular Contact Topology, Lattice Distortion, and Excited-State Relaxation

To understand the origin of the narrower emission, distinct decay dynamics, and reduced PLQY observed for MA­(18C6), temperature-dependent PL measurements were performed between 10 and 300 K (Figure S8a–c). All compounds exhibit decreasing emission intensity and progressive spectral broadening with increasing temperature, consistent with thermally activated nonradiative relaxation mediated by lattice phonons.

The strength of the exciton–phonon coupling was evaluated from the temperature dependence of the emission line width (FWHM), using the following expression^42,43^

$$\mathrm{FWHM}\;\left(T\right)=2.36\sqrt{S}\cdot {\hbar \omega }_{\mathrm{phonon}}\sqrt{\coth\frac{{\hbar \omega }_{\mathrm{phonon}}}{2k_{B}T}}$$

Here *S* is the Huang–Rhys factor, a dimensionless parameter that reflects the strength of the exciton–phonon coupling, ℏω_phonon_ is the effective phonon energy coupled to the electronic transition, *k*_B_ is the Boltzmann constant, and *T* is the absolute temperature. Fitting the experimental FWHM (*T*) data (Figure 3a,d,g), yields *S* = 84 and ℏω_phonon_ = 8.18 meV for NH_4_(18C6), *S* = 36 and ℏω_phonon_ = 15.6 meV for MA­(18C6), and *S* = 73, and ℏω_phonon_ = 9.3 meV for PEA­(18C6). All three compounds therefore fall within the regime of strong exciton–phonon coupling (*S* > 5).^14^ Nevertheless, the substantially lower Huang–Rhys factor of MA­(18C6) indicates weaker lattice relaxation in the excited state, consistent with its **∼**25% narrower emission bandwidth and suggesting a more rigid environment.

**Figure 3:**
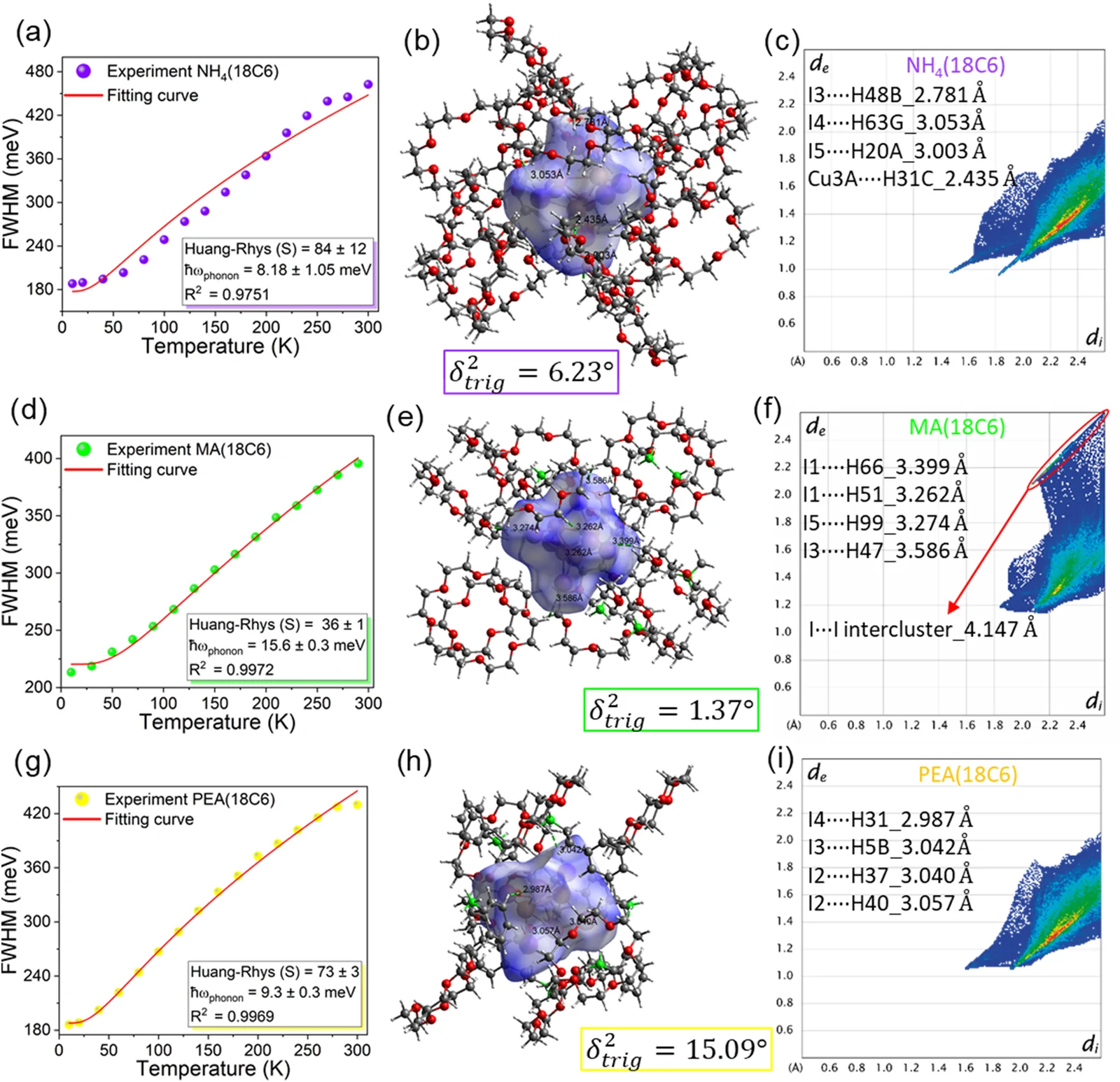
(a, d, g) Huang–Rhys fitting of the FWHM (T) data for the three compounds. (b, e, h) Calculated Hirshfeld surface for the respective [Cu_4_I_6_]^2–^ cluster showing I···H and Cu···H contacts and the resulting I–Cu–I trigonal bond angle variance (δ^2^). (c, f, i) 2D fingerplots for NH_4_(18C6), MA­(18C6) and PEA­(18C6) respectively.

To quantify this rigidity, the trigonal bond angle variance of the [CuI_3_]^2–^ coordination units within the [Cu_4_I_6_]^2–^ cluster was calculated using^44^

$$\delta_{\mathrm{trig}}^{2}=\frac{1}{n-1}\sum_{i=1}^{n}{\left(\theta_{i}-\theta_{120{}^{\circ}}\right)}^{2}$$

Where δ^2^ is the trigonal bond angle variance, θ_*i*_ is an individual I–Cu–I angle, and *n* is the number of angles considered. The resulting variances are 6.23°, 1.37°, and 15.09° for NH_4_(18C6), MA­(18C6), and PEA­(18C6), respectively, confirming that MA­(18C6) possesses the least distorted [Cu_4_I_6_]^2–^ cluster geometry. The I–Cu–I bond angles for each compound are presented in Tables S4–S6.

To probe the origin of these differences, Hirshfeld surface analysis was performed to visualize intermolecular contacts involving the [Cu_4_I_6_]^2–^ cluster anions.^44–46^ For NH_4_(18C6), the Hirshfeld surface (Figure 3b) reveals several short I···H and Cu···H contacts between the cluster and the surrounding organic framework. These contacts occur at distances ranging from 2.435 to 3.053 Å and appear as sharp spikes in the corresponding two-dimensional fingerprint plot (Figure 3c), indicating substantial host-cluster contact density.

In contrast, the Hirshfeld surface calculated for MA­(18C6) reveal negligible I···H or Cu···H contacts between the [Cu_4_I_6_]^2–^ clusters and the surrounding organic framework (Figure 3e). The corresponding contact distances range from 3.262 to 3.586 Å (Figure 3f), exceeding the sum of the relevant van der Waals radii. This absence of close host-cluster contacts indicates a comparatively weakly perturbed local environment, consistent with the smaller angular distortion and reduced exciton–phonon coupling of MA­(18C6).

For PEA­(18C6), the Hirshfeld surface again reveals numerous short contacts between the cluster and the surrounding organic framework. In particular, I···H and Cu···H contacts are observed with distances ranging from 2.987 to 3.057 Å (Figure 3h), and these appear as sharp features in the fingerprint plot (Figure 3i). Notably, PEA­(18C6) exhibits the largest angular distortion among the three compounds, likely induced by the spatially asymmetric distribution of hydrogen contacts imposed by the bulky phenethylammonium cation. The percentage contribution of each contact and the corresponding fingerprint plots for each compound are provided in Table S7 and Figures S9–S11.

Taken together, these results demonstrate that distortion of the [Cu_4_I_6_]^2–^ cluster is governed by the crystal packing imposed by the organic supramolecular component. Ultimately, this packing defines the nature, topology, and spatial distribution of the host-cluster interactions. Systems exhibiting numerous short, anisotropically distributed hydrogen-mediated contacts, such as NH_4_(18C6) and PEA­(18C6), impose uneven local constraints on the [Cu_4_I_6_]^2–^ cluster, leading to increased I–Cu–I bond angular variance, strengthening exciton–phonon coupling, and promoting lattice relaxation in the excited state. In contrast, the reduced contact density in MA­(18C6) yields a less perturbed cluster environment, consistent with its narrower emission.

Another notable feature of MA­(18C6) is a sharp region in the fingerprint plot at (*d*_i_; *d*_e_) = (2.1; 2.1), assigned to I···I intercluster contacts (Figure 3f) that are absent in the other two phosphors. These intercluster halogen contacts (Figure S12a,b) may account for the long-lived decay component (14.6 μs) observed in MA­(18C6) and could also contribute to its reduced PLQY. These results reveal how variations in the supramolecular environment are reflected in both structural distortion and excited-state behavior.

### Electronic Structure and Exciton Confinement

Because MA­(18C6) and PEA­(18C6) define the two clearest end points of the supramolecular tuning series, minimal cluster distortion versus enhanced distortion, respectively. DFT calculations were performed to examine how these structural differences influence electronic confinement. The calculated electronic band structures (Figure 4a,b) show that the conduction band minimum (CBM) of MA­(18C6) is significantly more dispersive, whereas PEA­(18C6) exhibits much flatter conduction and valence bands, reflecting weaker electronic coupling between neighboring cluster units in PEA­(18C6).

**Figure 4:**
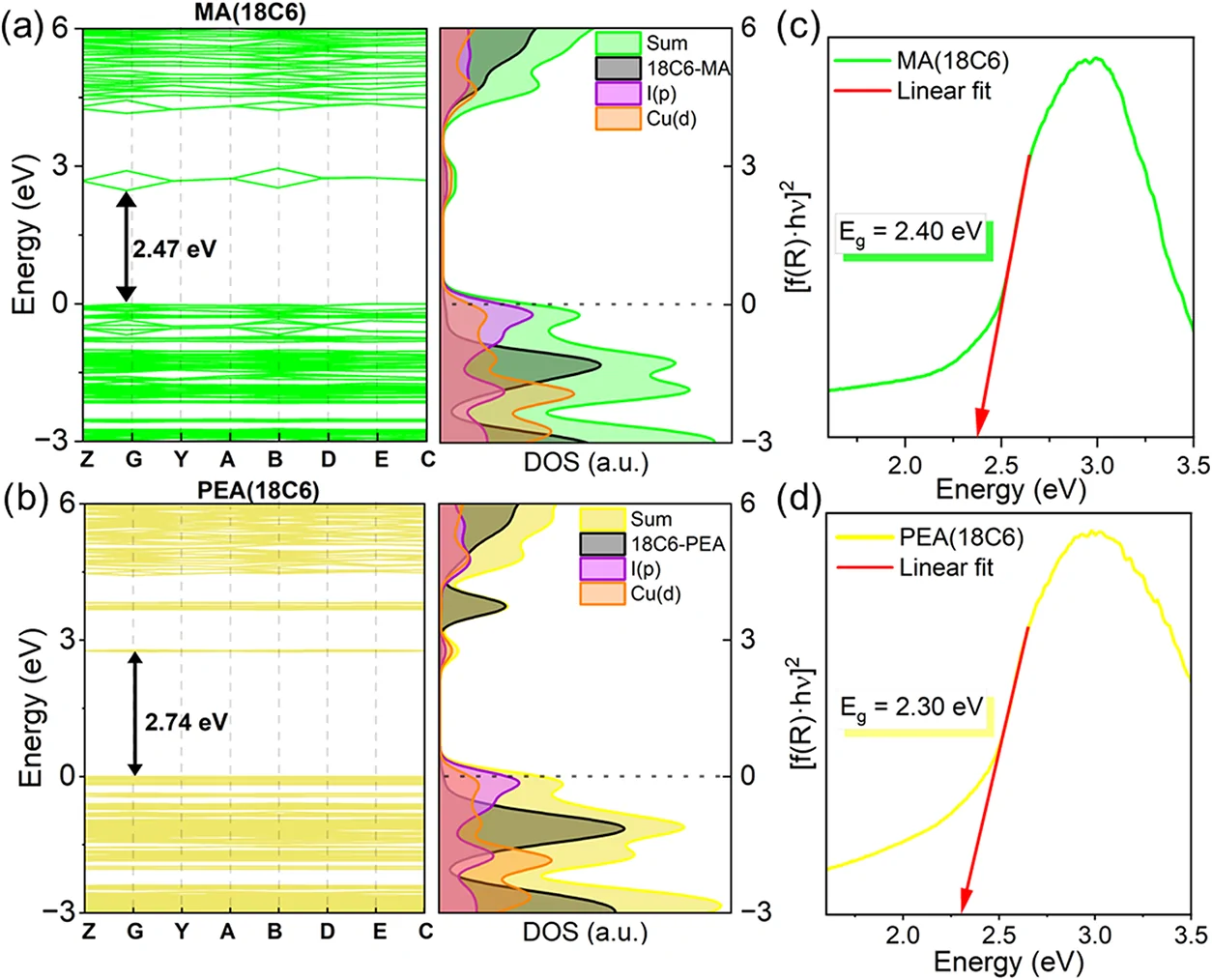
(a–b) Calculated electronic band structure and partial/total density of states for MA­(18C6) and PEA­(18C6). (c–d) Experimental bangaps extracted from the Tauc plot of the diffuse reflectance spectra for the two phosphors.

Analysis of the density of states (DOS) for both compounds reveals that the band-edge states are strongly localized within the [Cu_4_I_6_]^2–^ clusters, consistent with the electronically zero-dimensional nature of the cluster array inferred from the crystal structures. These states are predominantly composed of Cu 3d and I 5p orbitals (Figure 4a,b), suggesting that the supramolecular framework does not contribute to the electronic states at the band edges. Accordingly, the differences in band dispersion are attributed to variations in electronic interactions between neighboring clusters, likely associated with differences in their spatial arrangement imposed by the supramolecular environment.

Exciton binding energies (*E*_b_) were estimated from temperature-dependent PL measurements by fitting an Arrhenius model to the integrated emission intensity as a function of temperature (Figure S13a,b). The obtained values are *E*_b_ = 93.72 meV for MA­(18C6) and *E*_b_ = 57.6 meV for PEA­(18C6). These relatively high values are consistent with strong excitonic confinement within the [Cu_4_I_6_]^2–^ clusters, in agreement with the localized character of the band-edge states inferred from the DOS analysis.

In this context, the stronger band dispersion observed for MA­(18C6) suggests increased electronic delocalization near the band edges, whereas the flatter bands of PEA­(18C6) are consistent with a higher degree of electronic confinement within the [Cu_4_I_6_]^2–^ clusters, consistent with the enhanced localization of the corresponding Cu 3d and I 5p states in the DOS. These results support a cluster-centered electronic structure in which the band-edge states are largely confined to the [Cu_4_I_6_]^2–^ units. Although photoluminescence efficiency is ultimately governed by the balance between radiative and nonradiative decay pathways, this increased electronic confinement is expected to favor exciton localization and radiative recombination within the cluster units, providing a plausible explanation for the higher PLQY observed for PEA­(18C6). Conversely, the greater band dispersion in MA­(18C6) may facilitate additional relaxation pathways, which could contribute to the lower PLQY and the long-lived decay component observed experimentally.

The calculated band gaps do not follow the experimental trend obtained from diffuse reflectance measurements (Figure 4c,d), which can be attributed to the well-known delocalization error associated with GGA-PBE exchange–correlation functionals.^47^ These findings provide insight into how supramolecular interactions influence the electronic structure and emission properties.

### Environmental Stability and Phosphor Converter Performance

From a practical perspective, it is also important to evaluate the environmental stability and device performance of these materials. Considering that converter phosphors in phosphor-converted white LEDs typically operate at temperatures between ∼60 and 120 °C^48^ the thermal robustness of the compounds was evaluated by TGA (Figure 5a). NH_4_(18C6) exhibits the lowest thermal stability, with a decomposition onset temperature of 123 °C, whereas MA­(18C6) and PEA­(18C6) show improved thermal robustness, with degradation onsets around 145 °C. Variable-temperature PXRD measurements (RT–110 °C) reveal temperature-dependent variations in peak intensities for all compounds (Figure S14). Among them, MA­(18C6) exhibits the most consistent diffraction profile, indicative of enhanced structural rigidity upon thermal perturbation and consistent with the reduced structural distortion of the [Cu_4_I_6_]^2–^ clusters.

**Figure 5:**
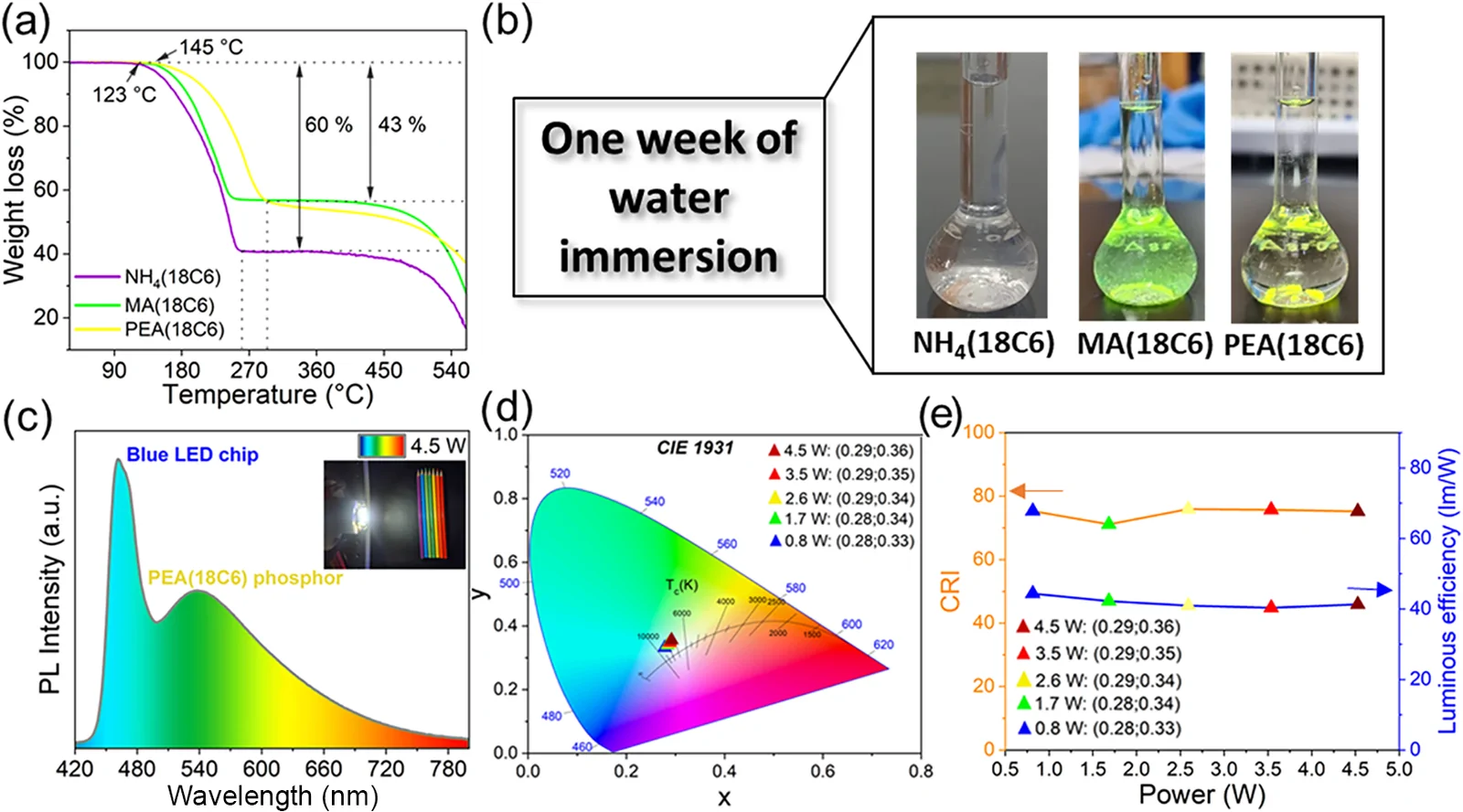
(a) Thermogravimetric analysis of the three compounds. (b) Photographs of the compounds after 1 week of water immersion, showing luminescence after that time. (c) Emission spectrum of the fabricated pc-WLED, with an inset photograph of the operating device. (d) CIE 1931 chromaticity coordinates of the pc-WLED emission under different applied powers. (e) Variation of the color rendering index (CRI) and luminous efficiency of the pc-WLED as a function of applied power.

Water stability was also evaluated because moisture sensitivity remains a major limitation for many metal halide phosphors.^49^ MA­(18C6) and PEA­(18C6) exhibit excellent resistance to moisture, retaining their structural integrity after 1 week of immersion. In contrast, NH_4_(18C6) shows pronounced degradation, as evidenced by visual inspection (Figure 5b) and PXRD patterns collected before and after water exposure (Figure S15).

Owing to its combined thermal and moisture stability, together with its broadband emission, PEA­(18C6) was selected for phosphor-converter applications. A video demonstrating its rapid, solvent-free mechanochemical synthesis under 450 nm illumination is provided in the Supporting Information, showing the rapid formation of the luminescent phosphor within approximately 2 min. The mechanochemically synthesized phosphor exhibits PL and PLE spectra identical to those of the solution synthesized material (Figure S16a), and its powder XRD pattern matches the simulated pattern calculated from the single-crystal structure (Figure S16b). These results further demonstrate the practical accessibility of this material. Its integration onto a 450 nm blue LED yielded an efficient pc-WLED. The emission spectra of the fabricated pc-WLED is shown in Figure 5c.

The CIE chromaticity coordinates show only minor power-dependent variations, ranging from (0.28; 0.33) to (0.29; 0.36), and remains close to white emission (Figure 5d). The resulting light can be classified as cold white because of the limited red component in the spectrum. Nevertheless, the CRI approaches 80, which is notable considering the absence of a dedicated red emitter. The luminous efficacy exceeds 40 lm·W^–1^, remaining nearly constant across the applied power range (Figure 5e).

These results demonstrate that supramolecular copper halide cluster phosphors can combine strong broadband emission with improved environmental stability, together with the simplicity and scalability of the mechanochemical synthesis. PEA­(18C6) represents not only a promising material for sustainable phosphor-converter systems for solid-state lighting, but also highlights the broader potential of supramolecular host–guest control over copper halide clusters. By stabilizing the [Cu_4_I_6_]^2–^ unit through crown-ether mediated coordination of onium-type cations, this strategy provides a modular handle to tune the local environment of the emissive cluster. In this context, the separation between the emissive metal halide unit and the supramolecular host framework establishes a platform in which the optical and environmental properties of the material can be systematically tuned through chemical modification of the surrounding organic components while preserving the underlying cluster motif. Such a design principle opens a pathway toward the rational development of copper halide phosphors with tailored emission characteristics, stability, and processability for optoelectronic applications.

## Conclusions

In conclusion, this work establishes a supramolecular strategy for stabilizing and tuning emissive [Cu_4_I_6_]^2–^ copper­(I) iodide clusters using 18-crown-6 in combination with ammonium-derived cations of increasing steric and hydrophobic character. Across the NH_4_^+^, CH_3_NH_3_^+^, and phenethylammonium series, systematic variation of the supramolecular cation environment modulates lattice disorder, water incorporation, and the degree of distortion of the [Cu_4_I_6_]^2–^ cluster while preserving the underlying cluster motif. Structural analysis together with Hirshfeld surface mapping reveals that host–cluster contacts, primarily involving H···I and H···Cu interactions, define the intermolecular contact topology surrounding the inorganic cluster and correlate with the extent of cluster distortion.

These structural variations translate directly into changes in the excited-state response. Increased angular distortion of the [Cu_4_I_6_]^2–^ unit is associated with stronger exciton–phonon coupling and broader emission, whereas the weaker perturbed cluster environment in MA­(18C6) yields reduced lattice relaxation and a narrower emission profile. Density functional theory calculations further support a cluster-centered electronic structure in which the band-edge states remain strongly localized within the [Cu_4_I_6_]^2–^ units, consistent with the electronically zero-dimensional nature of these materials.

Beyond clarifying the structure–property relationships governing this family of supramolecular copper halides, the present results identify PEA­(18C6) as a promising phosphor-converter material that combines broadband visible emission, improved thermal and moisture stability, and efficient white pc-WLED performance, all achieved through rapid, solvent-free mechanochemical synthesis. More broadly, this work demonstrates that supramolecular host–guest design provides a modular platform for engineering copper-halide cluster phosphors. By decoupling the emissive inorganic cluster from the surrounding organic framework, this strategy enables systematic tuning of optical response, environmental stability, and processability through chemical modification of the supramolecular environment, thereby expanding the design space for sustainable copper-halide phosphors for solid-state lighting and related optoelectronic applications.

## Supplementary Material





## Notes

Mechanochemical PEA­(18C6) (MP4)
Powder X-ray diffraction characterization for all compounds, additional structural details, information about intermolecular contacts, low-temperature photoluminescence characterizations and TD-DFT calculations (PDF)
