# Supplementary material for: A Crown Ether-Ammonium Supramolecular Platform for Luminescent [Cu4I6]2– Cluster Phosphors
Source: ACS Phys Chem Au. 2026 Jul 24;6(5):1320–30. doi: 10.1021/acsphyschemau.6c00098 (PMC13614131; doi:10.1021/acsphyschemau.6c00098)
Supplement: Supplementary file 1 [file pg6c00098_si_002.pdf]

**Supplementary information**

# A Crown Ether-Ammonium Supramolecular Platform for Luminescent $[Cu_4I_6]^{2-}$ Cluster Phosphors

*Disnel Ferrera-Carracedo, *[a] Vojtech Jancik, [b, c] Estrella Ramos, [a] Diego Solis-Ibarra, [a] Juan de Dios Guzmán-Hernández, [b, c] J C. Alonso-Huitron, [a] Ioantani D. Álvarez, [a] Jesús U. Balderas-Aguilar. *[a]*

*email of corresponding authors: disfer_96@comunidad.unam.mx, jubalderas@materiales.unam.mx*

[a] Instituto de Investigaciones en Materiales, Universidad Nacional Autónoma de México, Ciudad Universitaria, A.P. 70-360, Coyoacán, Ciudad de México, México.

[b] Instituto de Química, Universidad Nacional Autónoma de México, Ciudad Universitaria, Ciudad de México 04510, México.

[c] Centro Conjunto de Investigación en Química Sustentable UAEM-UNAM, Carretera Toluca-Atlacomulco km. 14.5, Toluca, Estado de México 50200, México.

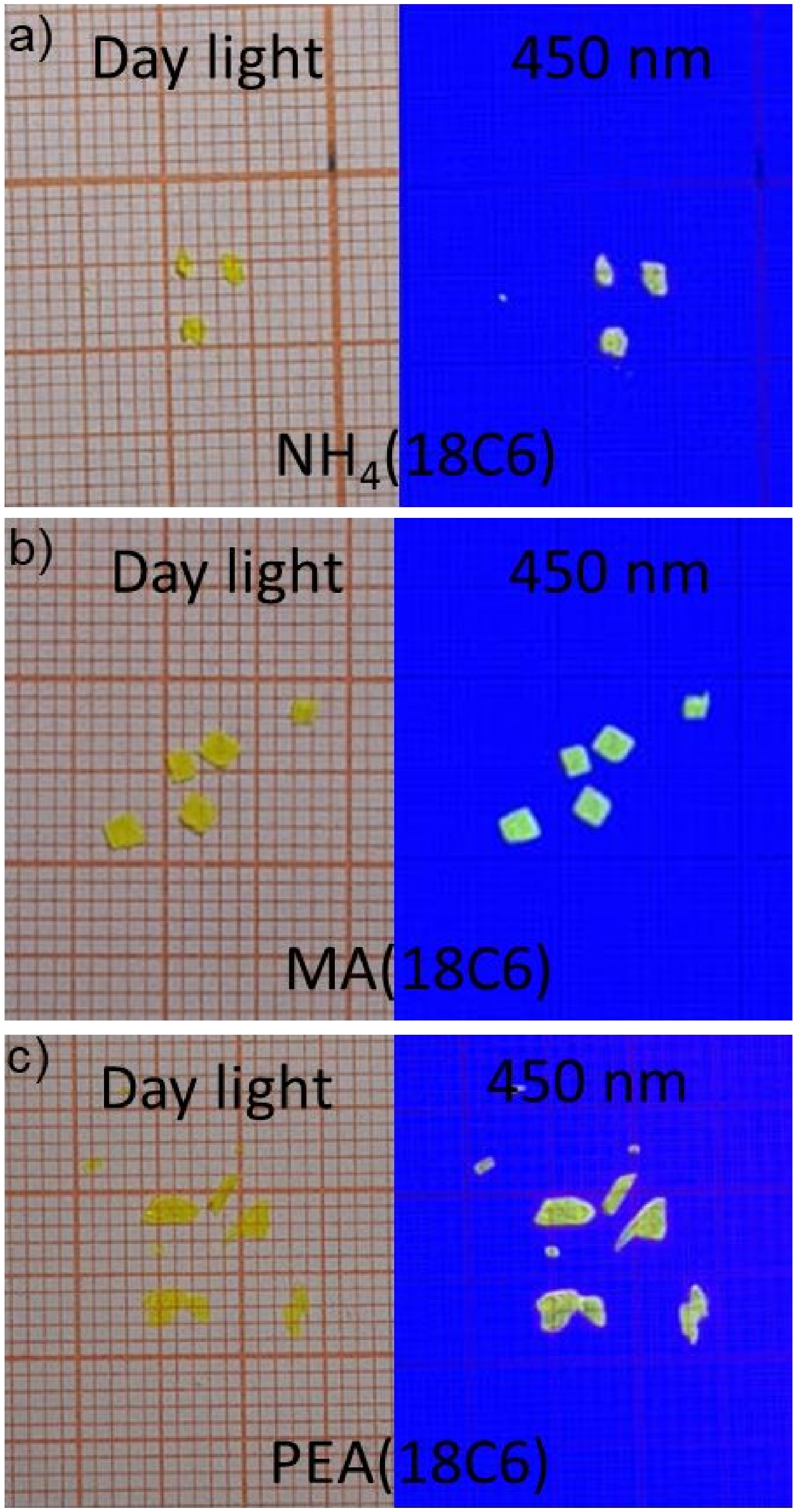


**Figure S1.** (a–c) Synthesized compounds $NH_4$(18C6), MA(18C6), and PEA(18C6) under daylight and 450 nm blue light illumination.

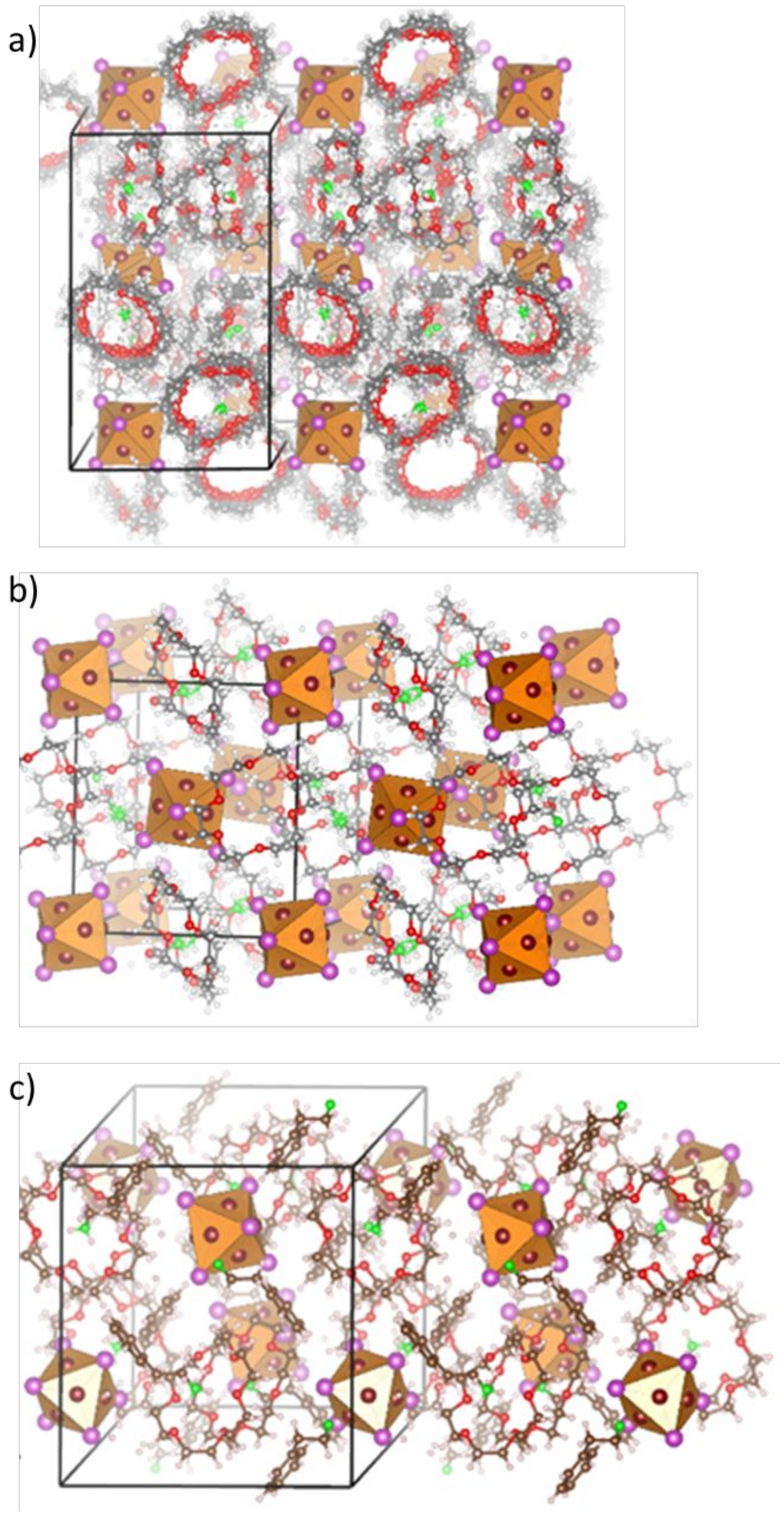


**Figure S2.** (a–c) Crystalline structure models of the three synthesized compounds with their corresponding unit cells, shown from top to bottom: $NH_4$(18C6), MA(18C6), and PEA(18C6).

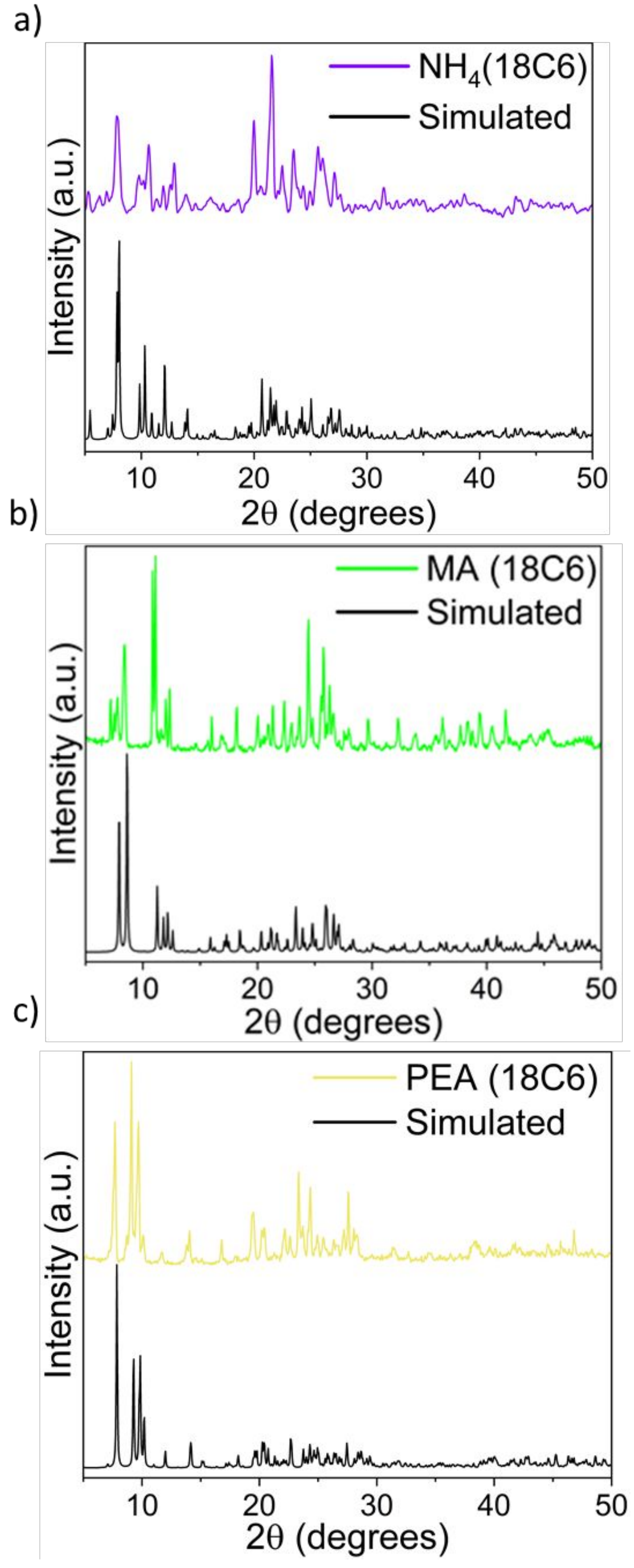


**Figure S3.** (a–c) Experimental powder X-ray diffraction patterns compared with the simulated ones for each synthesized compound.

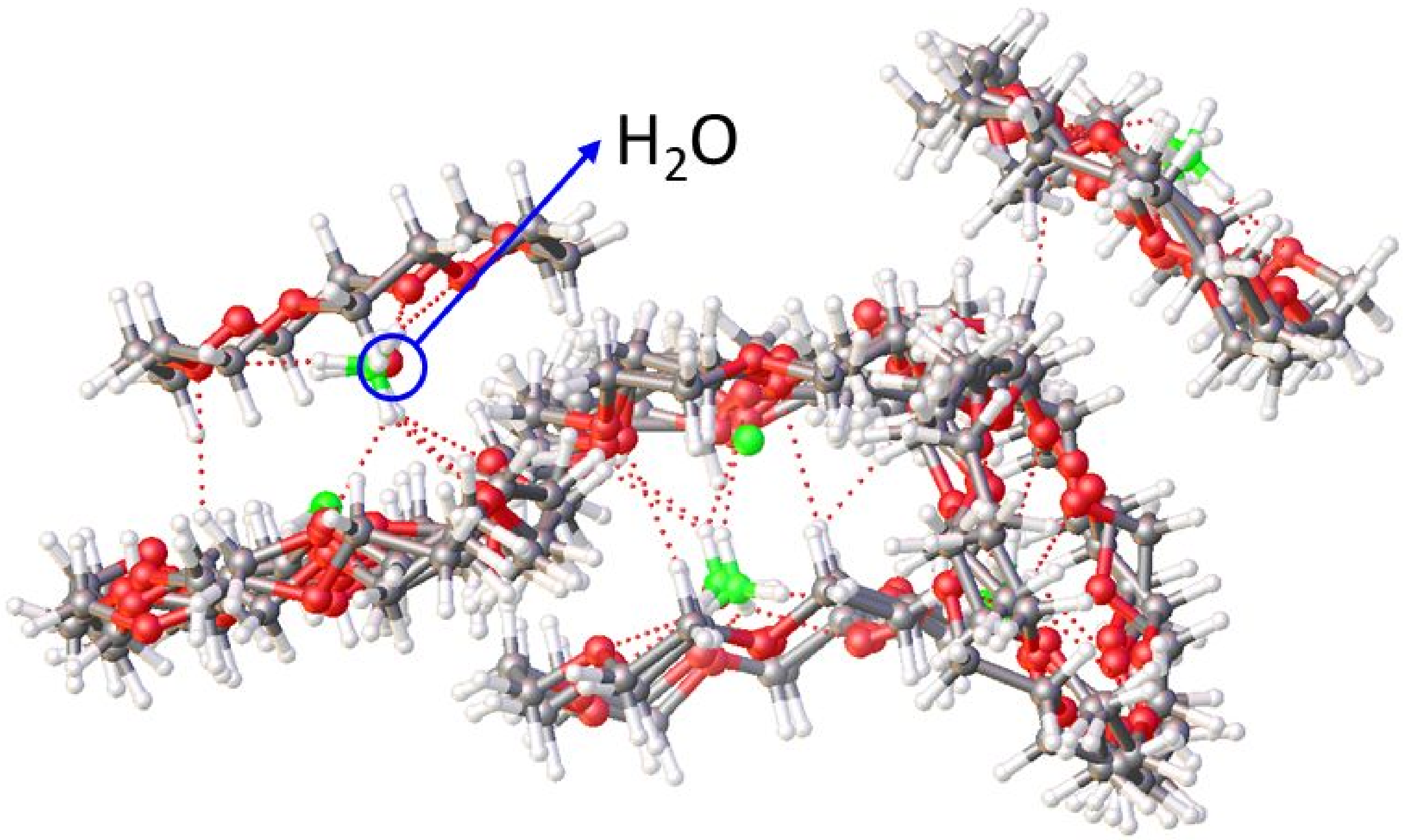


**Figure S4.** Organic moiety of the $NH_4$(18C6) compound showing the hydrogen-bonding interactions. The N–H···O and C–H···O contacts are highlighted with red dotted lines. The small $NH_4^+$ cation acts as a bridge between two 18-crown-6 molecules, contributing to the high degree of structural disorder.

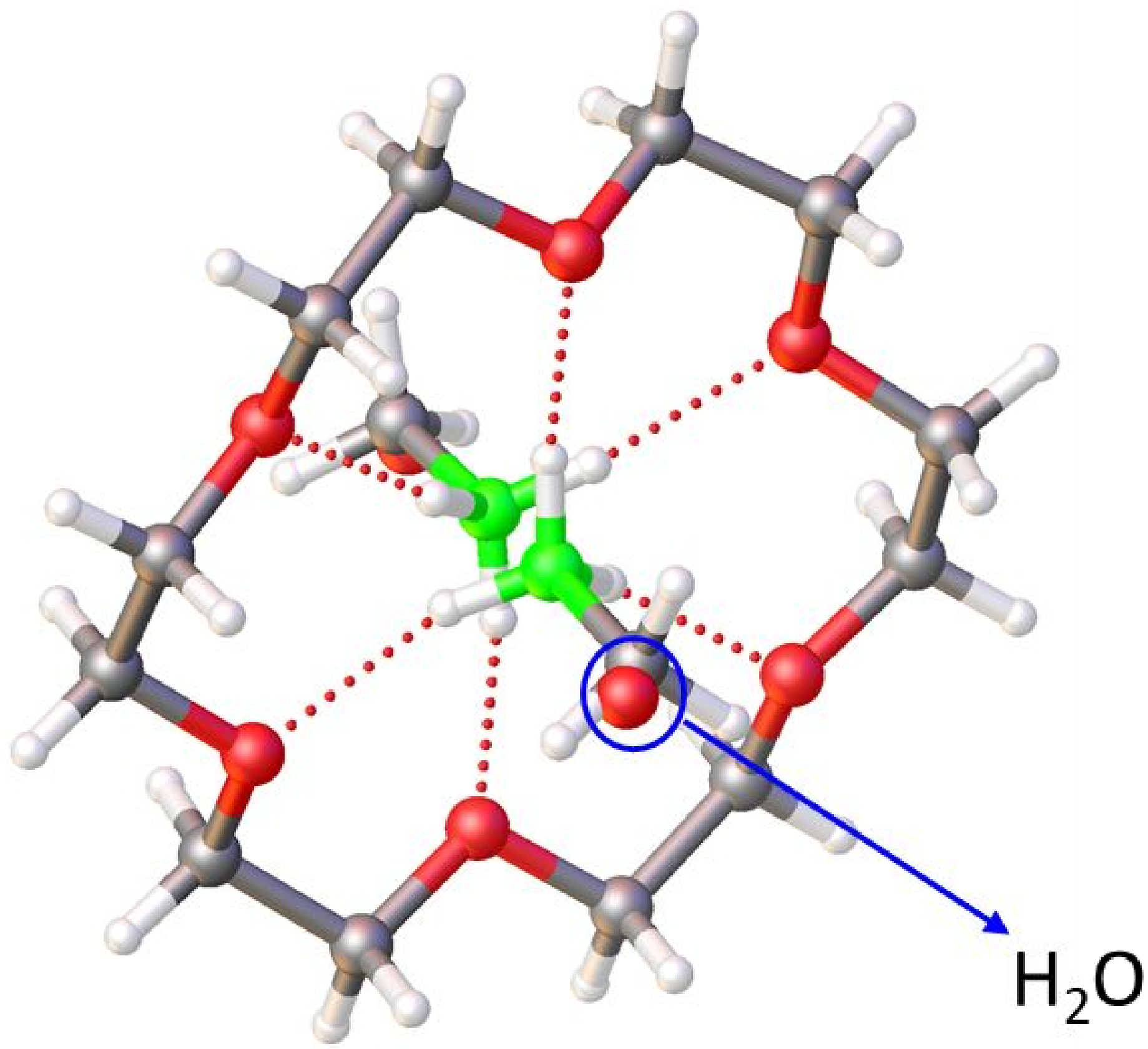


**Figure S5.** Organic moiety of the MA(18C6) compound showing the hydrogen-bonding interactions N···H···O highlighted in dotted red lines. The methylammonium cation is disordered over two positions on each side of the 18C6 molecule.

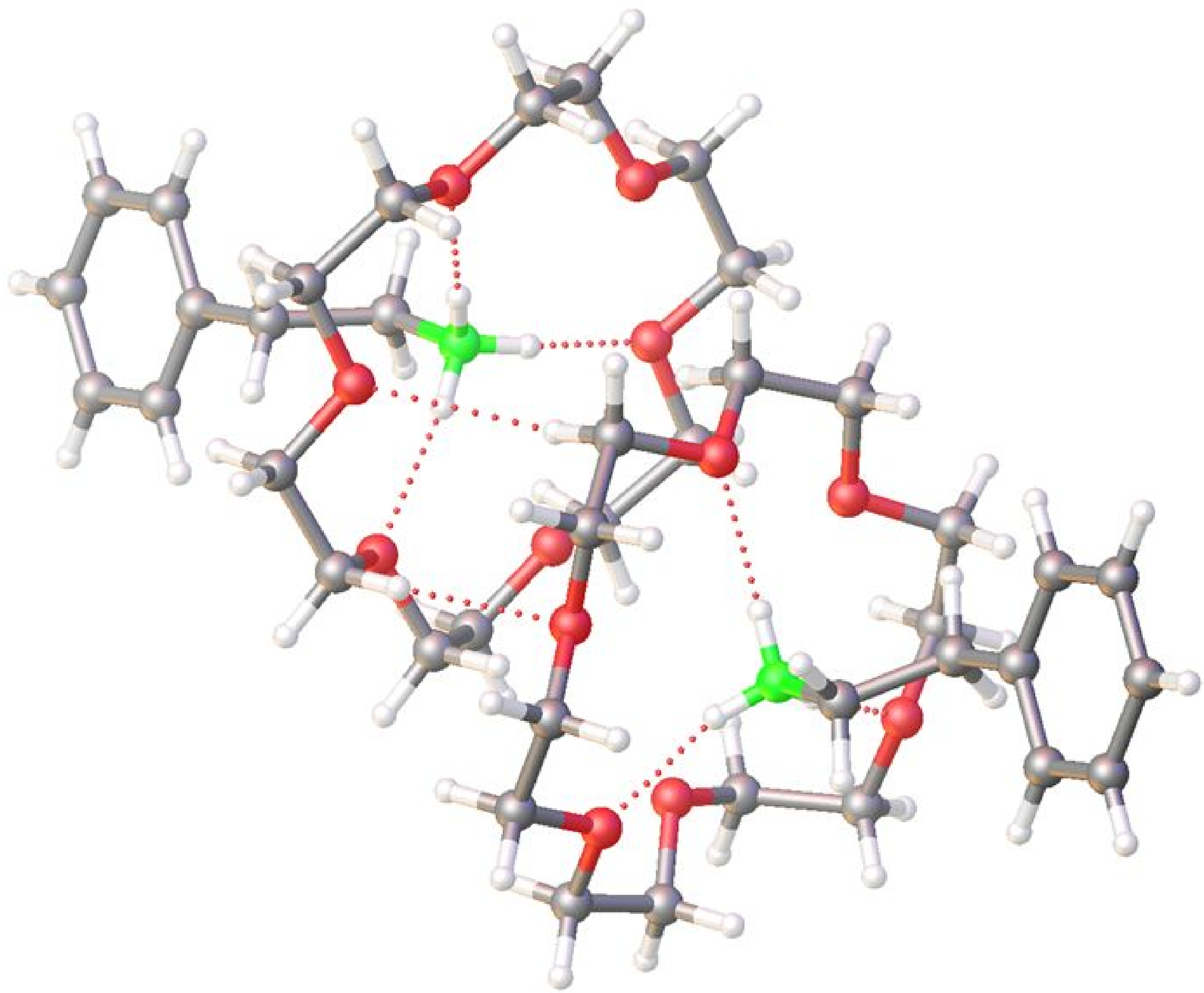

**Figure S6.** Organic moiety of the PEA(18C6) compound showing the hydrogen-bonding interactions N···H···O highlighted in dotted red lines. There is no disorder and no water incorporation within the structure.

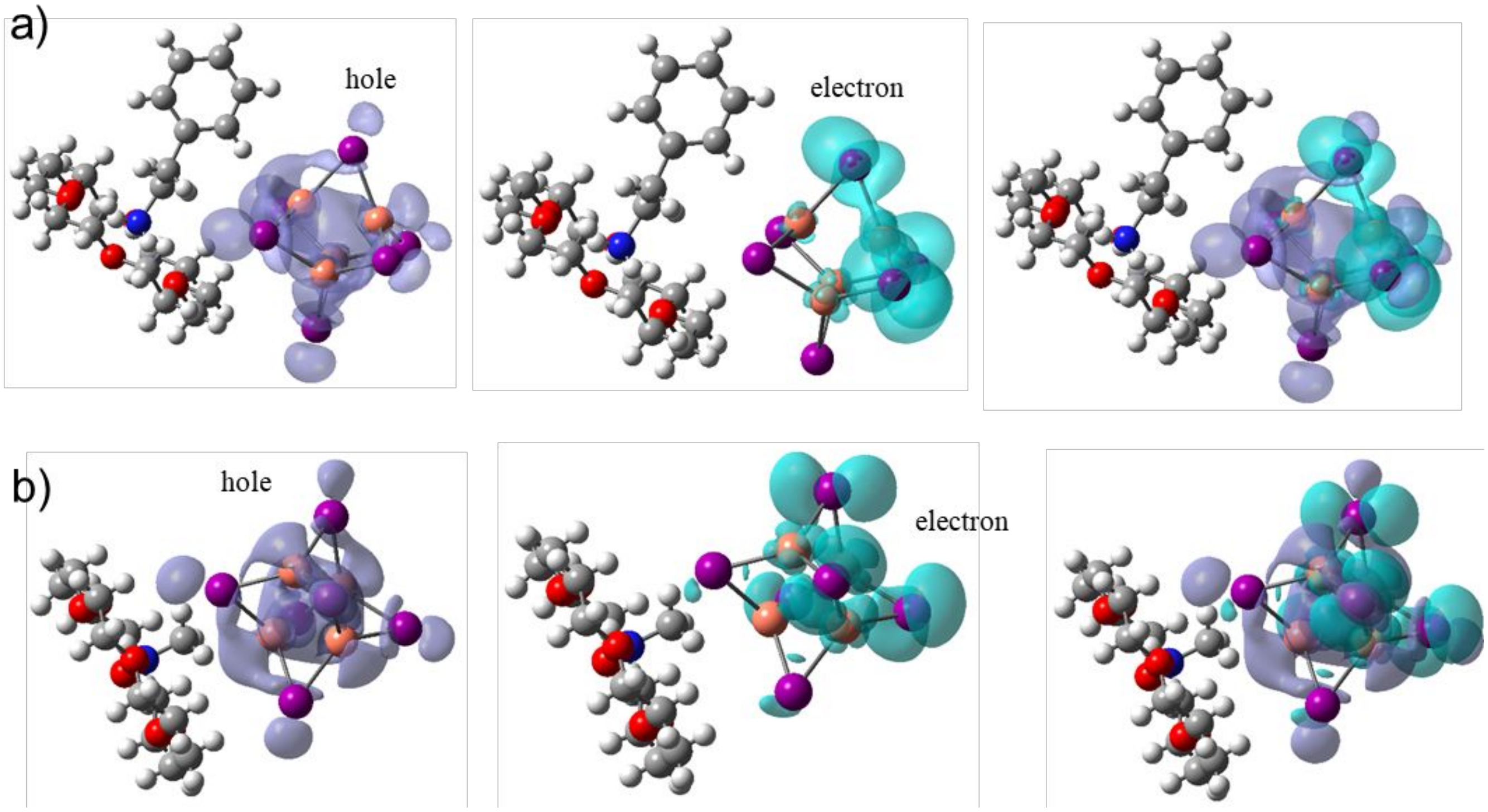


**Figure S7**. Charge Density Difference (CDD) maps obtained from TD-DFT calculations of the first excited state for (a) PEA(18C6) and (b) MA(18C6).

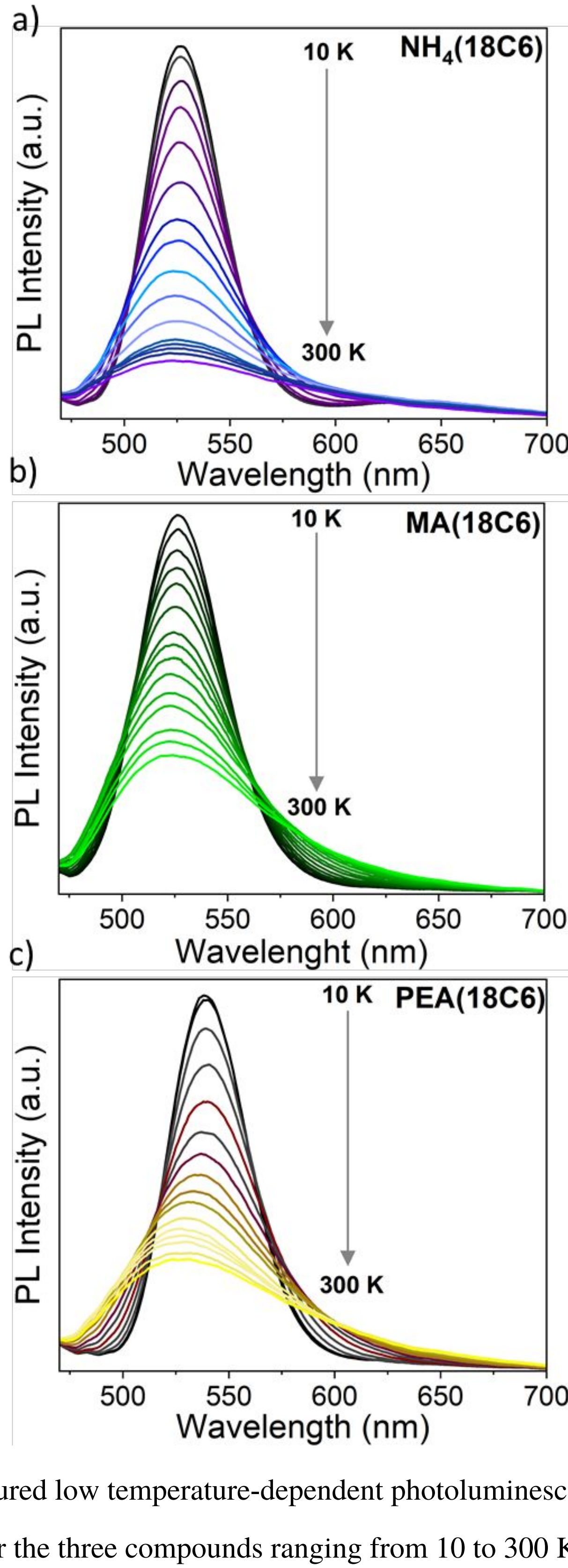


**Figure S8.** (a–c) Measured low temperature-dependent photoluminescence emission spectra for the three compounds ranging from 10 to 300 K.

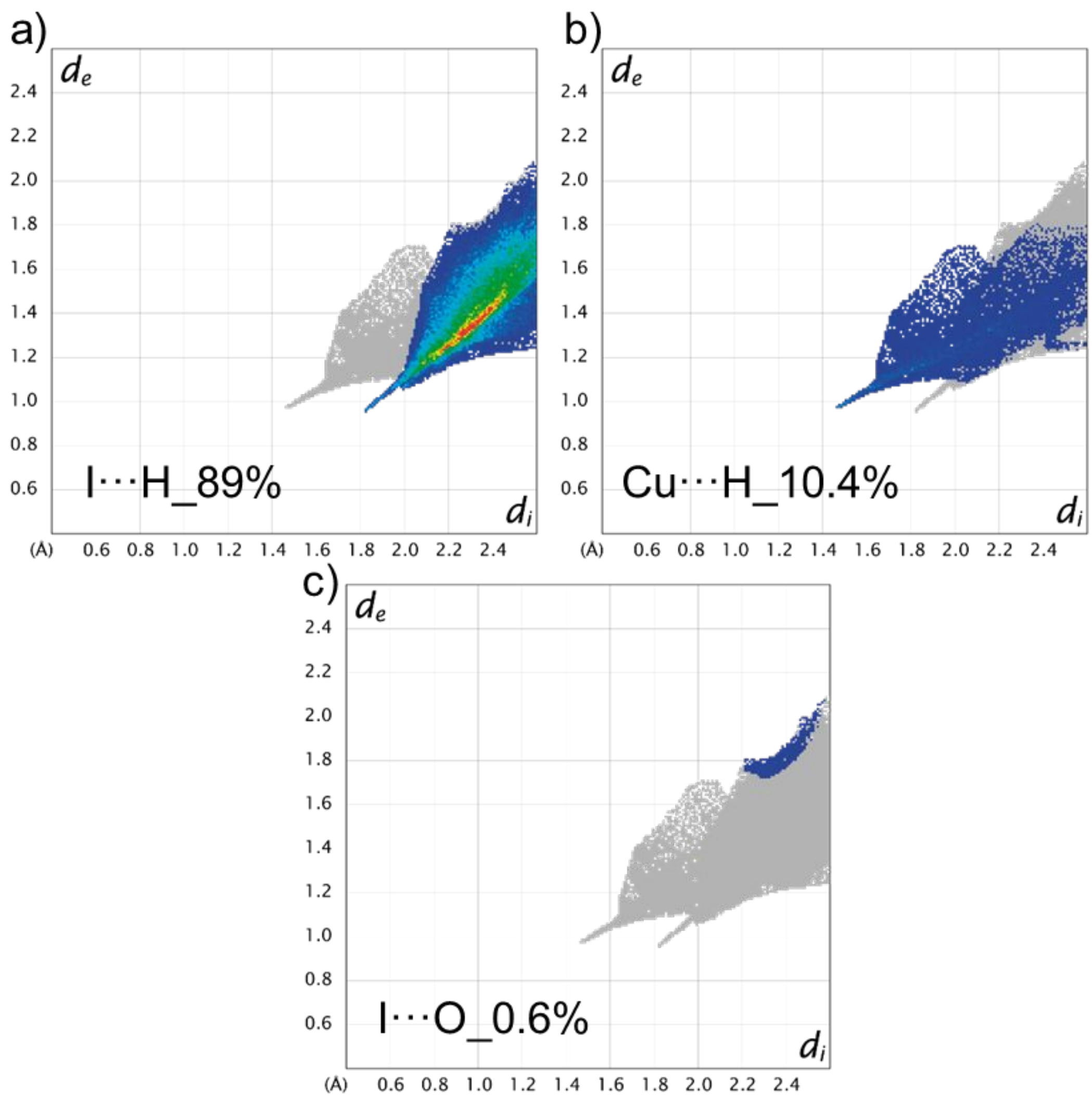


**Figure S9.** (a–c) Percentage contribution of each intermolecular interaction in the fingerprint plots for $NH_4$(18C6), describing contacts between the $[Cu_4I_6]^{2-}$ cluster and the organic framework.

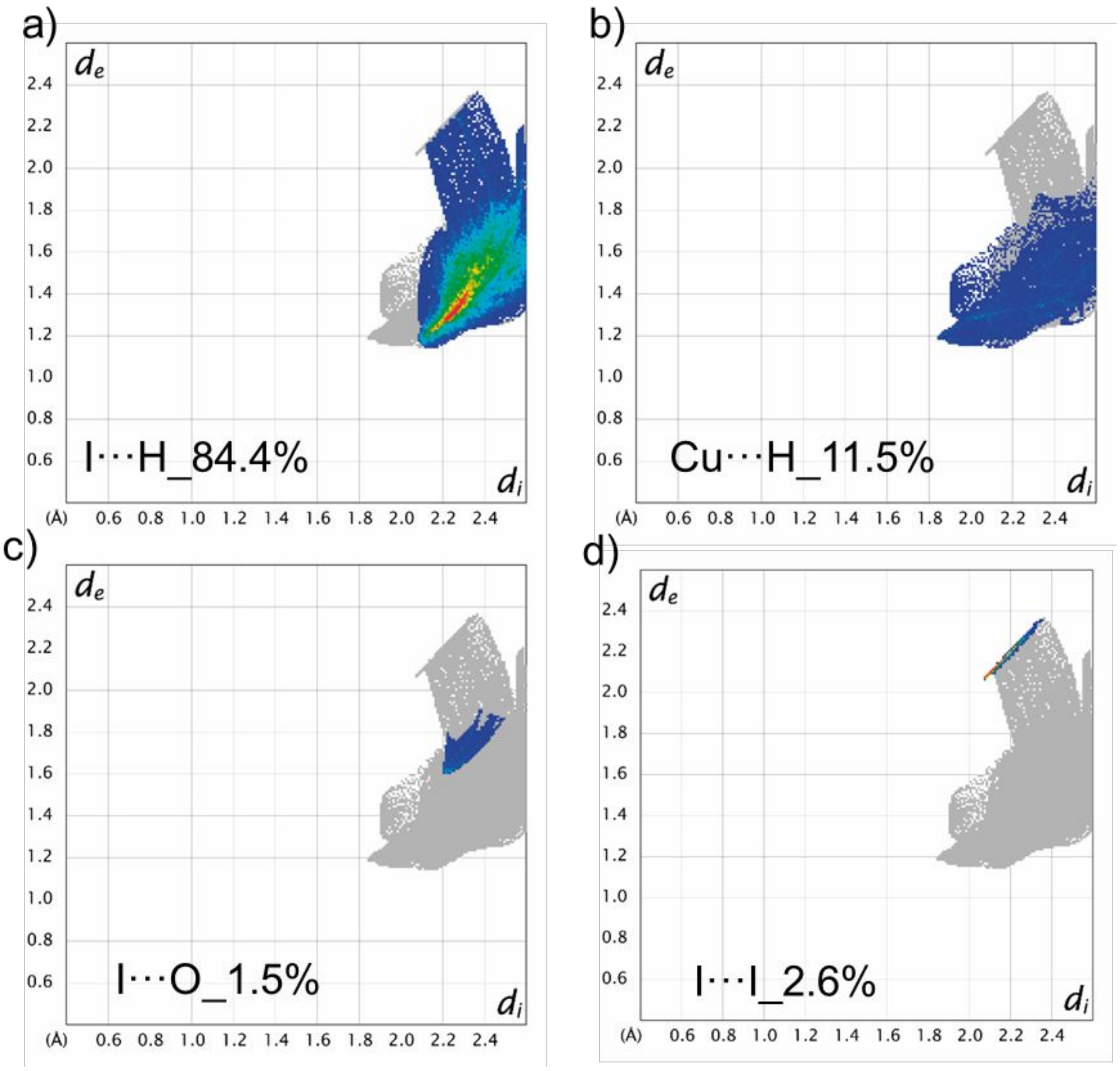


**Figure S10.** (a–e) Percentage contribution of each intermolecular interaction in the fingerprint plots for MA(18C6), describing contacts between the $[Cu_4I_6]^{2-}$ cluster and the organic framework.

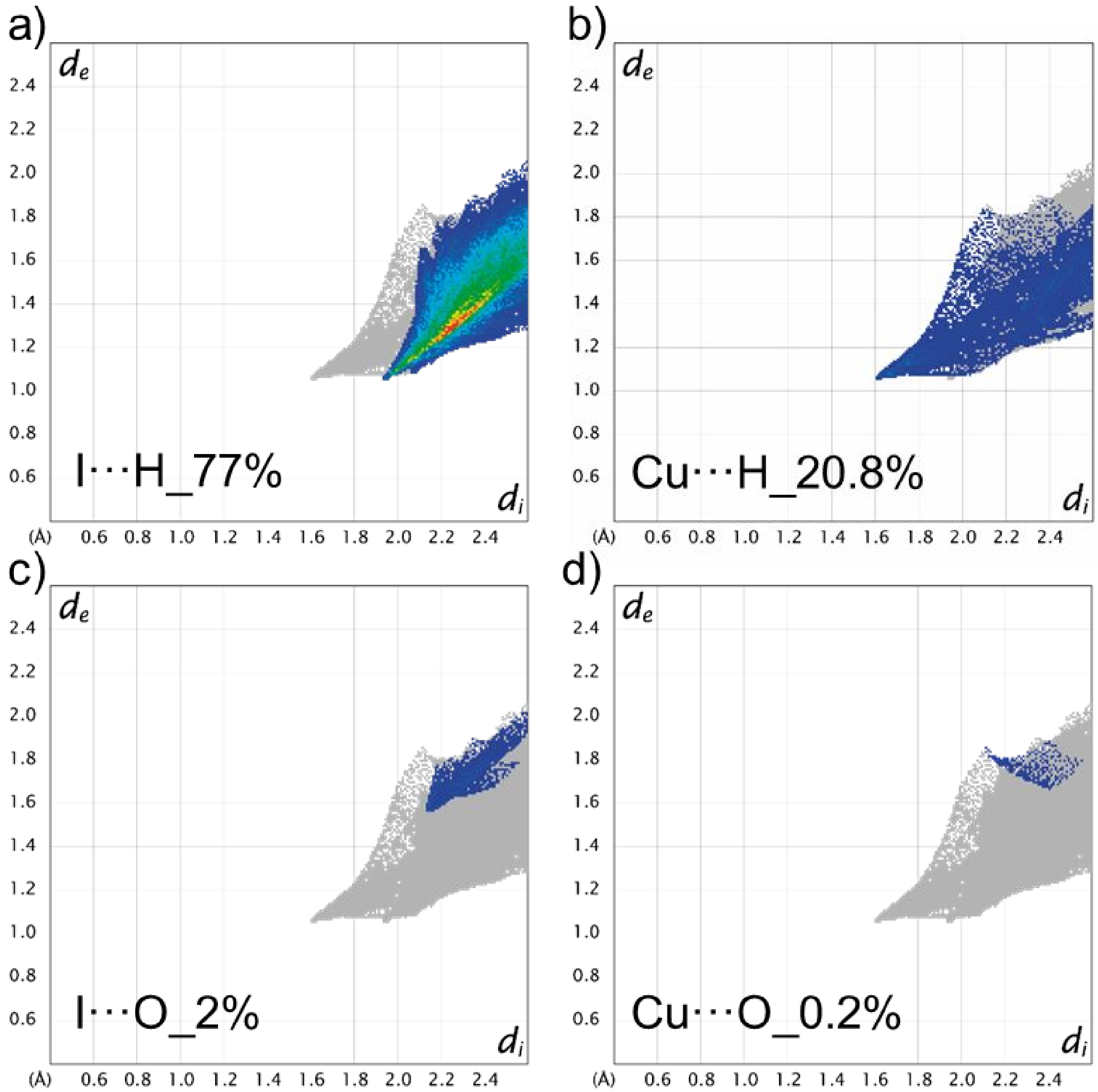


**Figure S11.** (a–d) Percentage contribution of each intermolecular interaction in the fingerprint plots for PEA(18C6), describing contacts between the $[Cu_4I_6]^{2-}$ cluster and the organic framework.

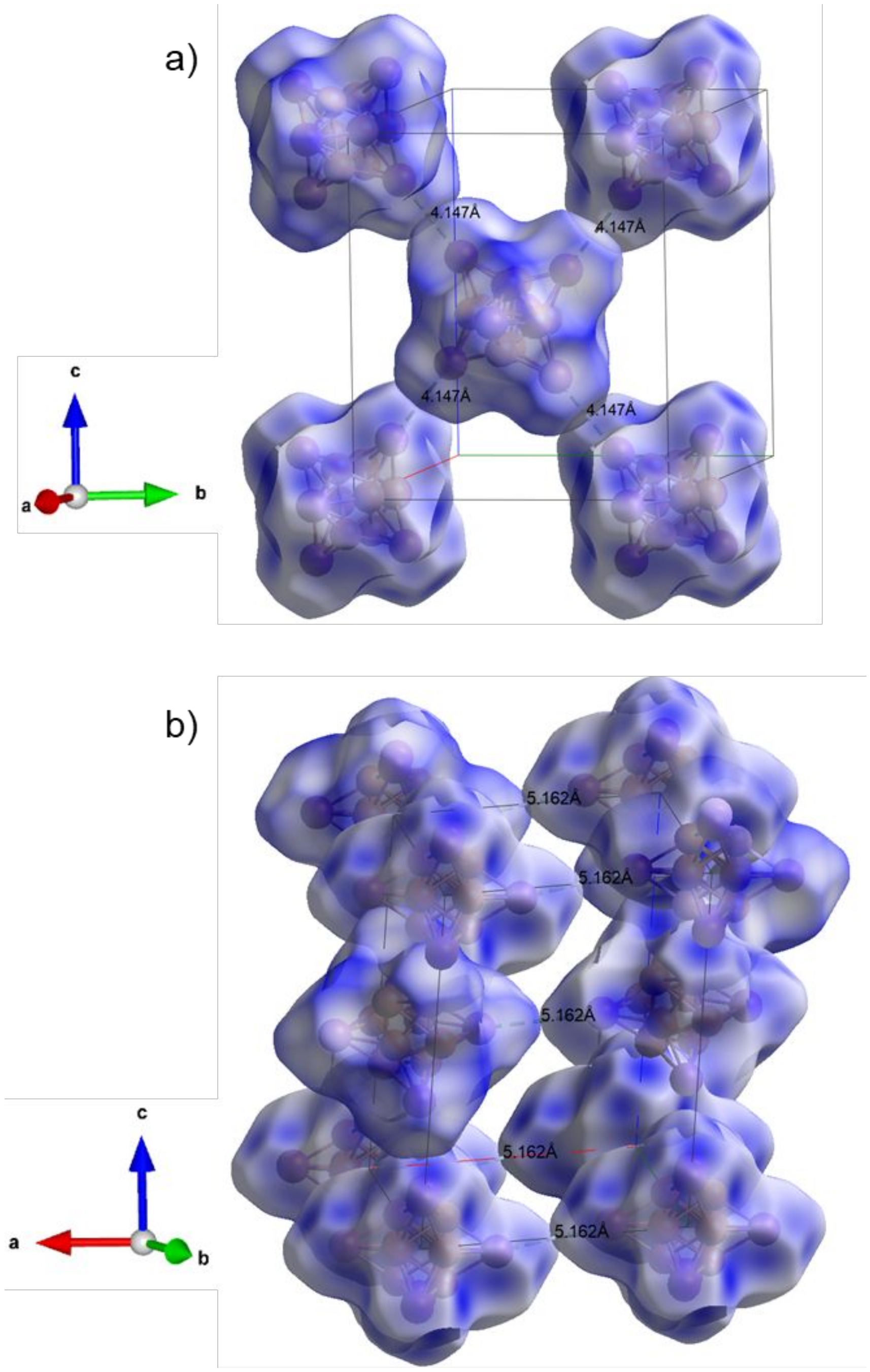


**Figure S12.** (a) Hirshfeld surfaces of $[Cu_4I_6]^{2-}$ clusters in MA(18C6) showing I···I intercluster contacts along the diagonals in the bc plane of the unit cell. (b) Hirshfeld surfaces on the same compound with a rotated view along the plane ac showing no I···I intercluster interactions, the organic network has been omitted for clarity.

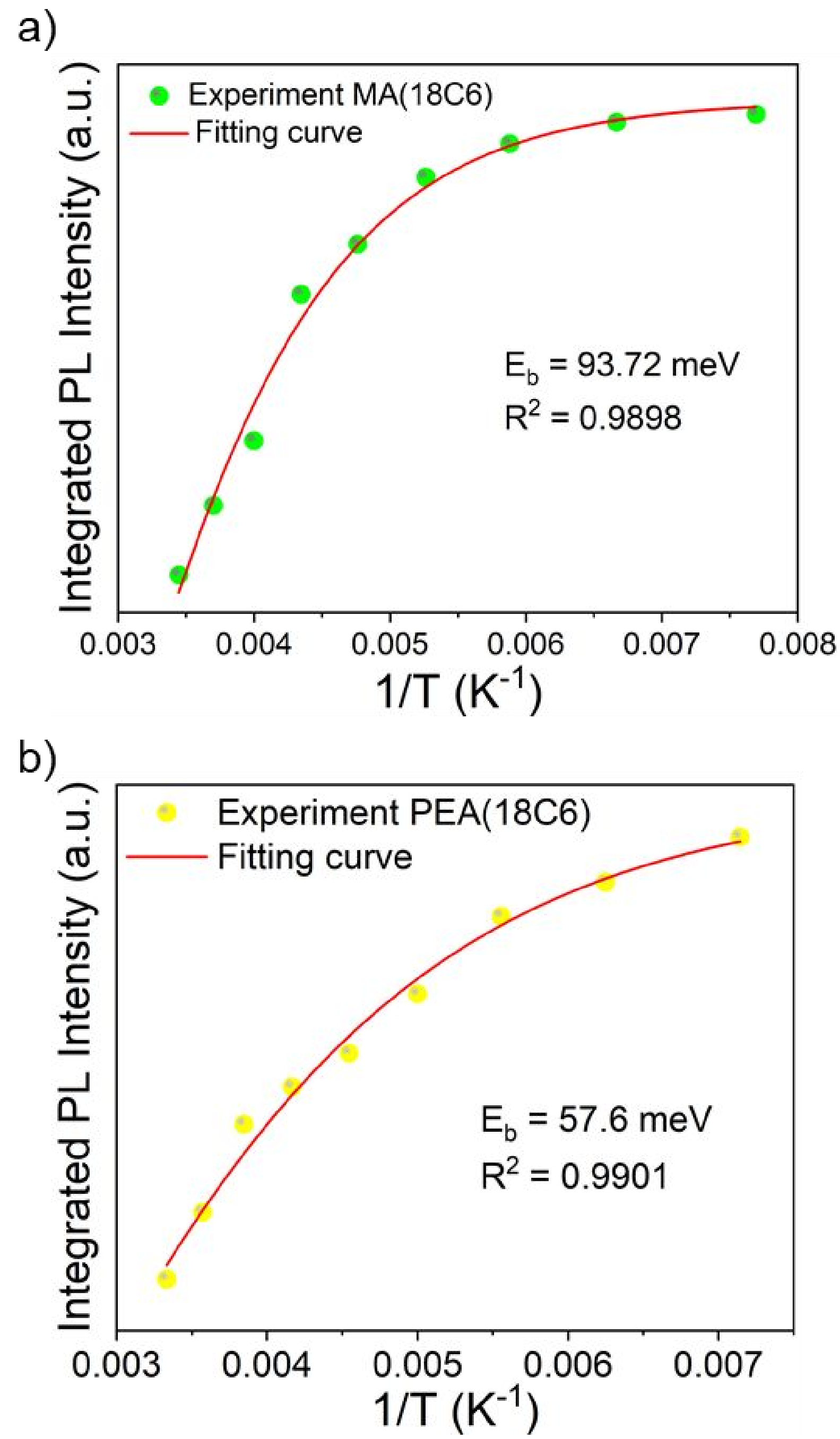


**Figure S13.** (a, b) Extracted $E_b$ values for MA(18C6) and PEA(18C6) respectively obtained from the Arrhenius model.

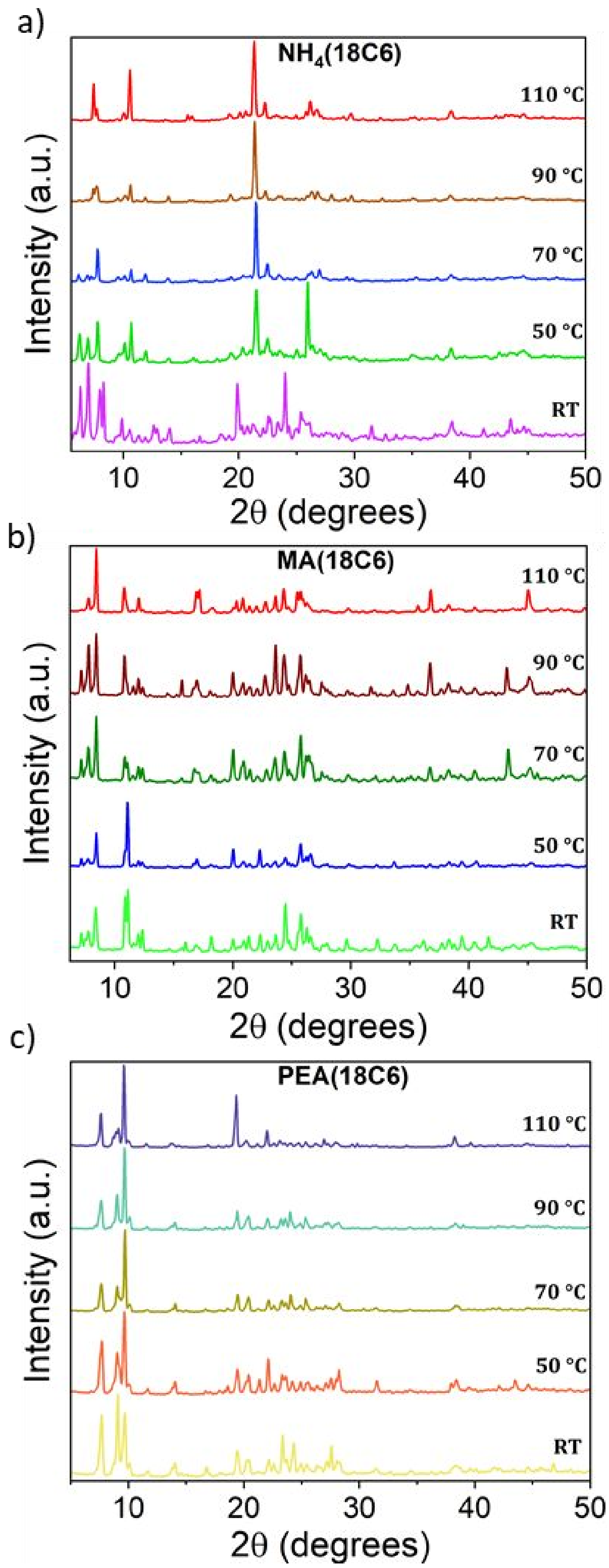


**Figure S14.** (a–c) Temperature-dependent PXRD for the three compounds $NH_4$(18C6), MA(18C6) and PEA(18C6) respectively.

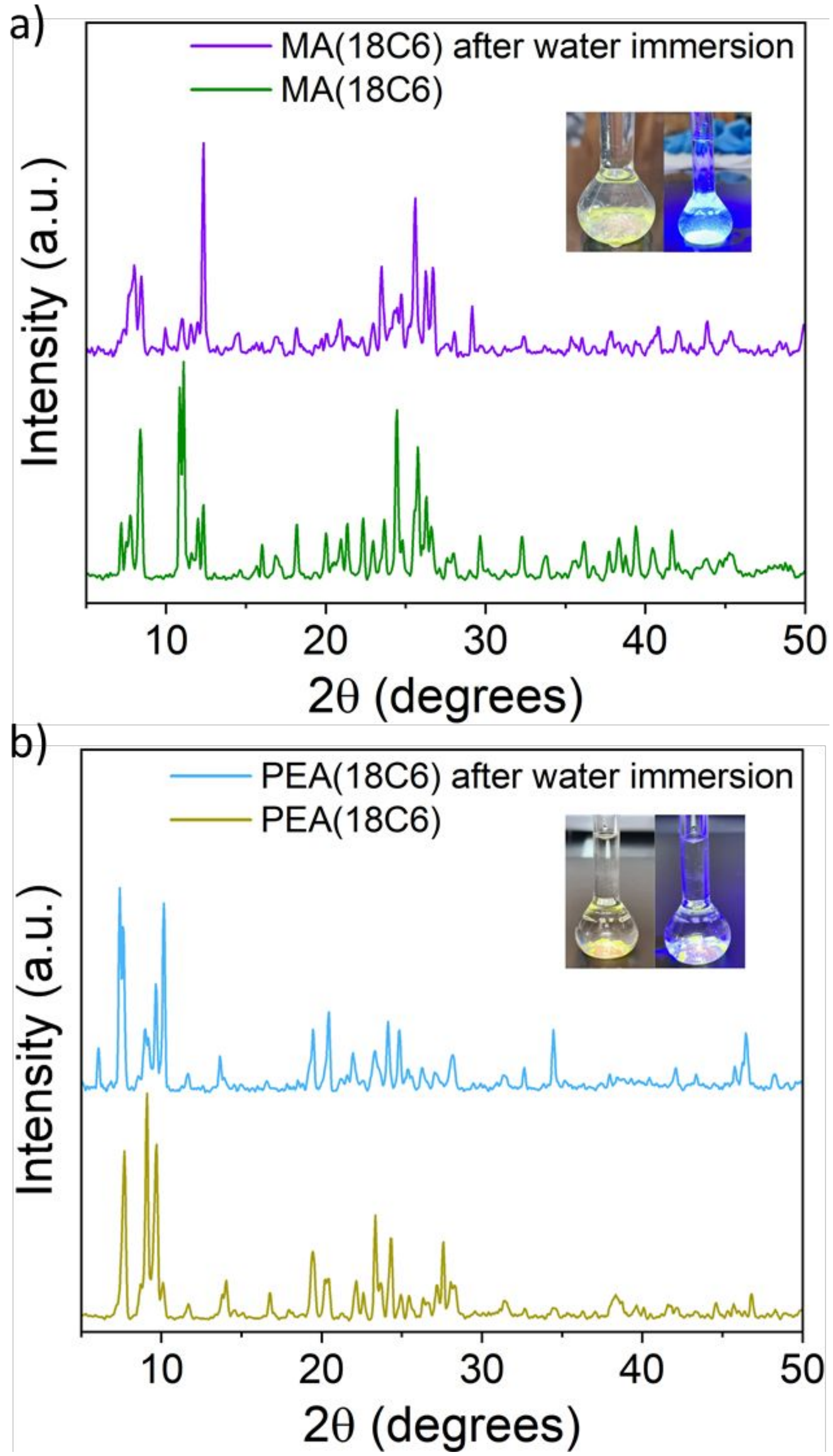


**Figure S15.** (a, b) PXRD before and after water immersion for MA(18C6) and PEA(18C6), respectively.

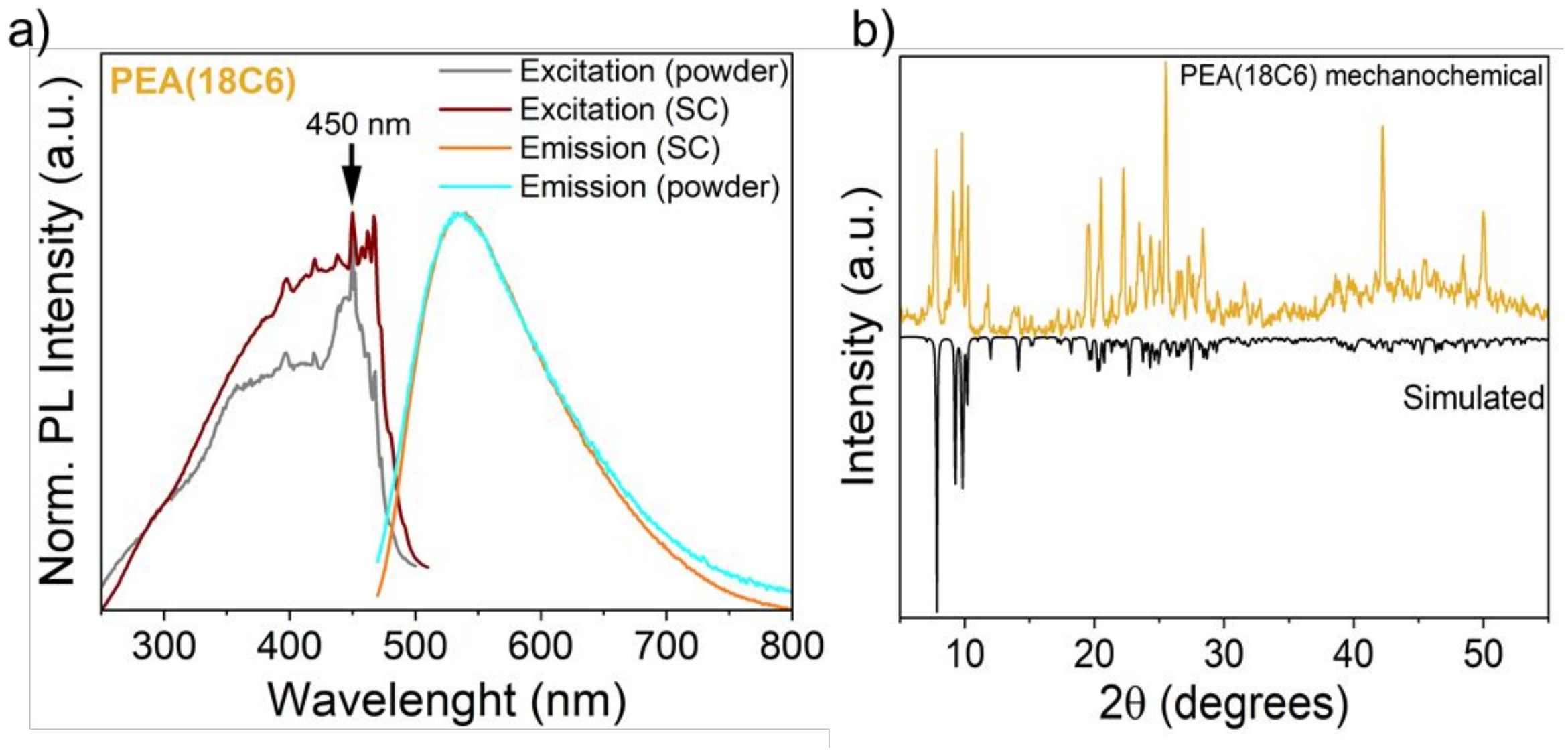


**Figure S16.** (a) Comparison of the PLE an PL emission spectra of PEA(18C6) single crystals and the corresponding powders obtained by mechanochemical synthesis. (b) XRD diffraction pattern of PEA(18C6) powders obtained from mechanochemical synthesis.

**Table S1.** Crystal data and structure refinement details for **$NH_4$(18C6):**

| | |
|---|---|
| formula | C72 H160.51 Cu6 I10 N4 O36.25 |
| moiety formula | 6(C12 H24 O6), Cu4 I6, Cu2 I4, 4(H4 N), |
| 0.506(H2 O) | |
| fw | 3312.83 |
| crystal system | monoclinic |
| space group | P n |
| temp, K | 100(2) |
| $\lambda$, Å | 0.71073 |
| $a$, Å | 18.5500(6) |
| $b$, Å | 13.9267(5) |
| $c$, Å | 23.3108(8) |
| $\alpha$, deg | 90 |
| $\beta$, deg | 104.7110(10) |
| $\gamma$, deg | 90 |
| $V$, Å$^3$ | 5824.7(3) |
| $Z$ | 2 |
| $\rho$, g·cm$^{-3}$ | 1.889 |
| $\mu$, mm$^{-1}$ | 3.796 |
| $F$(000) | 3229 |
| crystal size, mm$^3$ | 0.303 x 0.178 x 0.124 |
| $\theta$ range for data collection, deg | 1.462 to 33.213 |
| index ranges | $-28 \leq h \leq 28$ |
| | $-21 \leq k \leq 21$ |
| | $-35 \leq l \leq 35$ |
| no. of reflns collected | 187387 |
| no. of indep. reflns ($R_{int}$) | 44543 (0.0288) |
| no. of data / restraints / parameters | 44543 / 13700 / 3280 |
| GoF on $F^2$ | 1.027 |
| $R_1$,[a] $wR_2$[b] ($I > 2\sigma(I)$) | 0.0314, 0.0668 |
| $R_1$,[a] $wR_2$[b] (all data) | 0.0389, 0.0707 |
| abs. struct. par. | 0.497(14) |
| largest diff. peak / hole, e·Å$^{-3}$ | 2.605 / −2.131 |

[a] $R_1 = \sum||F_o| - |F_c||/\sum|F_o|$. [b] $wR_2 = [\sum w(F_o^2 - F_c^2)^2/\sum(F_o^2)^2]^{1/2}$.

**Table S2.** Crystal data and structure refinement details for **MA(18C6):**

| | |
|---|---|
| formula | C26 H60.65 Cu4 I6 N2 O12.33 |
| moiety formula | 2(C12 H24 O6), Cu4 I6, 2(C H6 N), 0.33(H2 O) |
| fw | 1614.20 |
| crystal system | monoclinic |
| space group | P 21/c |
| temp, K | 100(2) |
| $\lambda$, Å | 0.71073 |
| $a$, Å | 11.2197(3) |
| $b$, Å | 14.0397(4) |
| $c$, Å | 15.0932(4) |
| $\alpha$, deg | 90 |
| $\beta$, deg | 96.5400(10) |
| $\gamma$, deg | 90 |
| $V$, Å$^3$ | 2362.03(11) |
| $Z$ | 2 |
| $\rho$, g·cm$^{-3}$ | 2.270 |
| $\mu$, mm$^{-1}$ | 5.747 |
| $F$(000) | 1527 |
| crystal size, mm$^3$ | 0.502 x 0.198 x 0.122 |
| $\theta$ range for data collection, deg | 2.333 to 33.211 |
| index ranges | $-17 \le h \le 17$ |
| | $-21 \le k \le 21$ |
| | $-23 \le l \le 23$ |
| no. of reflns collected | 139148 |
| no. of indep. reflns ($R_{int}$) | 9056 (0.0315) |
| no. of data / restraints / parameters | 9056 / 73 / 287 |
| GoF on $F^2$ | 1.162 |
| $R_1$,[a] $wR_2$[b] ($I > 2\sigma(I)$) | 0.0161, 0.0352 |
| $R_1$,[a] $wR_2$[b] (all data) | 0.0177, 0.0356 |
| largest diff. peak / hole, e·Å$^{-3}$ | 1.030 / −0.675 |

[a] $R_1 = \sum||F_o| - |F_c||/\sum|F_o|$. [b] $wR_2 = [\sum w(F_o^2 - F_c^2)^2/\sum(F_o^2)^2]^{1/2}$.

**Table S3.** Crystal data and structure refinement details for **PEA(18C6):**

| | |
|---|---|
| formula | C40 H72 Cu4 I6 N2 O12 |
| moiety formula | 2(C12 H24 O6), Cu4 I6, 2(C8 H12 N) |
| fw | 1788.55 |
| crystal system | monoclinic |
| space group | P 21/c |
| temp, K | 100(2) |
| $\lambda$, Å | 0.71073 |
| $a$, Å | 18.7284(5) |
| $b$, Å | 17.3670(5) |
| $c$, Å | 18.5467(5) |
| $\alpha$, deg | 90 |
| $\beta$, deg | 104.6930(10) |
| $\gamma$, deg | 90 |
| $V$, Å$^3$ | 5835.2(3) |
| $Z$ | 4 |
| $\rho$, g·cm$^{-3}$ | 2.036 |
| $\mu$, mm$^{-1}$ | 4.664 |
| $F$(000) | 3424 |
| crystal size, mm$^3$ | 0.485 x 0.469 x 0.306 |
| $\theta$ range for data collection, deg | 1.624 to 33.270 |
| index ranges | $-28 \leq h \leq 28$ |
| | $-26 \leq k \leq 26$ |
| | $-28 \leq l \leq 28$ |
| no. of reflns collected | 157371 |
| no. of indep. reflns ($R_{int}$) | 22406 (0.0544) |
| no. of data / restraints / parameters | 22406 / 154 / 632 |
| GoF on $F^2$ | 1.056 |
| $R_1$,[a] $wR_2$[b] ($I > 2\sigma(I)$) | 0.0360, 0.0783 |
| $R_1$,[a] $wR_2$[b] (all data) | 0.0458, 0.0819 |
| largest diff. peak / hole, e·Å$^{-3}$ | 2.524 / −1.282 |

[a] $R_1 = \sum||F_o| - |F_c||/\sum|F_o|$. [b] $wR_2 = [\sum w(F_o^2 - F_c^2)^2/\sum(F_o^2)^2]^{1/2}$.

**Tables S4–S6.** I–Cu–I bond angles for the three compounds

| *Atoms ($NH_4$(18C6))* | $θ_i$ |
|---|---|
| *I1-Cu1-I4* | 118.48 |
| *I4-Cu1-I5* | 124.2 |
| *I1-Cu1-I5* | 115.69 |
| *I1-Cu2-I2* | 122.97 |
| *I1-Cu2-I3* | 116.32 |
| *I2-Cu2-I3* | 119.78 |
| *I3-Cu3-I4* | 120.65 |
| *I3-Cu3-I6* | 119.96 |
| *I4-Cu3-I6* | 119.07 |
| *I2-Cu4-I5* | 117.85 |
| *I2-Cu4-I6* | 120.23 |
| *I5-Cu4-I6* | 121.28 |

| *Atoms (MA(18C6))* | $θ_i$ |
|---|---|
| *I1-Cu1-I2* | 120.48 |
| *I1-Cu1-I3* | 120.92 |
| *I2-Cu1-I3* | 117.36 |
| *I1-Cu2-I2* | 121.52 |
| *I1-Cu2-I3* | 118.96 |
| *I2-Cu2-I3* | 118.96 |
| *I1-Cu3-I2* | 120.44 |
| *I1-Cu3-I3* | 118.74 |
| *I2-Cu3-I3* | 119.93 |
| *I1-Cu4-I2* | 119.34 |
| *I1-Cu4-I3* | 119.59 |
| *I2-Cu4-I3* | 120.34 |

| *Atoms (PEA(18C6))* | $θ_i$ |
|---|---|
| *I1-Cu1-I3* | 112.36 |
| *I1-Cu1-I4* | 123.52 |
| *I3-Cu1-I4* | 123.28 |
| *I1-Cu2-I2* | 117.75 |
| *I1-Cu2-I5* | 122 |
| *I2-Cu2-I5* | 119.67 |
| *I2-Cu3-I3* | 116.43 |
| *I2-Cu3-I6* | 117.35 |
| *I3-Cu3-I6* | 125.28 |
| *I4-Cu4-I5* | 117.3 |
| *I4-Cu4-I6* | 118.15 |
| *I5-Cu4-I6* | 124.12 |

**Table S7.** Percentage of intermolecular contacts for each compound extracted from Hirshfeld surface analysis.

| | **Percentage for each compound (%)** | | |
|---|---|---|---|
| *Intermolecular contact* | $NH_4$(18C6) | MA(18C6) | PEA(18C6) |
| *I···H* | 89 | 84.4 | 77 |
| *Cu···H* | 10.4 | 11.5 | 20.8 |
| *I···O* | 0.6 | 1.5 | 2 |
| *I···I (intercluster)* | | 2.6 | 0.2 |
